# Functional Outcomes and Ocular Safety of Subretinal AAV-RPGR Gene Therapy for RPGR-Associated X-Linked Retinitis Pigmentosa: A Systematic Review and Meta-Analysis

**DOI:** 10.3390/jcm15166144

**Published:** 2026-08-07

**Authors:** Carlos Roberto Montes-de-Oca-Saucedo, José Antonio Garza-Cruz, Bruno Briceño-Villardaga, Ingrid Vanessa Infante-Lee, Rafael Chavarría-Rojas, Natalie Carled Bermejo-Valero, Adolfo Soto-Domínguez, Neeran Narainswami

**Affiliations:** 1 Department of Histology, Faculty of Medicine, Universidad Autonoma de Nuevo Leon, Monterrey 64460, Mexico; antonio.garzacr@uanl.edu.mx (J.A.G.-C.); bruno.bricenov@uanl.edu.mx (B.B.-V.); ingrid.infantel@uanl.edu.mx (I.V.I.-L.); rc8453529@gmail.com (R.C.-R.); carled.bermejo@gmail.com (N.C.B.-V.); adolfo.sotodmn@uanl.edu.mx (A.S.-D.); 2 Division of Ophthalmology, Red Cross War Memorial Children’s Hospital and Groote Schuur Hospital, University of Cape Town, Cape Town 7700, South Africa; neeran.narainswami@uct.ac.za

**Keywords:** retinitis pigmentosa, RPGR, X-linked retinitis pigmentosa, adeno-associated virus, gene therapy, microperimetry, visual acuity, ocular safety, meta-analysis

## Abstract

**Background/Objectives**: We aimed to provide an updated synthesis of the efficacy and ocular safety of subretinal adeno-associated viral (AAV) RPGR gene therapy for retinitis pigmentosa GTPase regulator (RPGR)-associated X-linked retinitis pigmentosa (RPGR-XLRP) and to assess the certainty of the available evidence. **Methods**: We performed a PROSPERO-registered (CRD420251163589), PRISMA 2020 systematic review and meta-analysis using database and trial-registry searches. The primary microperimetry synthesis was restricted to mesopic Macular Integrity Assessment (MAIA) 68-loci acquisitions; continuous-outcome syntheses were conducted at the cohort level, using observed paired-eye contrasts when available; dose levels were interpreted within clinical programs; and above-maximum-tolerated-dose and peripheral-injection cohorts were reported separately. Random-effects analyses used DerSimonian–Laird models with Hartung–Knapp–Sidik–Jonkman small-sample correction. Risk of bias and certainty were assessed with design-matched tools and Grading of Recommendations Assessment, Development and Evaluation (GRADE). **Results**: The quantitative synthesis included four trials reported in five publications (128 participants; AAV8, AAV5, and AAV2tYF platforms). Mesopic retinal sensitivity numerically favored treatment, but the pooled differences were not statistically significant at six months (mean difference [MD] +1.11 dB; 95% confidence interval [CI], −0.88 to +3.09) or twelve months (MD +0.97 dB; 95% CI, −3.99 to +5.94). Best-corrected visual acuity showed no statistically significant pooled difference (MD +0.94 letters; 95% CI, −4.97 to +6.85). The six-month ≥10-letter low-luminance responder estimate favored treatment, but was nonsignificant and attenuated by twelve months; no primary pooled efficacy outcome reached significance under small-sample-adjusted inference. Certainty was very low for functional outcomes and low for safety outcomes. Uncontrolled treated-eye proportions were 80.8% for intraocular inflammation, 17.0% for ocular serious adverse events, and 4.3% for central-injection retinal detachment. **Conclusions**: Current evidence does not establish a clinically actionable functional benefit, although a modest true effect cannot be excluded. Clinically relevant ocular safety signals were observed, but the estimates were uncontrolled and program dependent. Adequately powered randomized trials with standardized functional endpoints, optimized subretinal delivery strategies, and careful within-program dose selection are needed.

## 1. Introduction

Retinitis pigmentosa GTPase regulator (RPGR)-associated X-linked retinitis pigmentosa (RPGR-XLRP) is one of the most severe inherited retinal degenerations, typically presenting with early-onset nyctalopia, progressive peripheral visual-field constriction, and eventual central visual impairment in affected males [1,2,3,4]. It remains an important cause of visual disability in working-age adults and a major unmet therapeutic need. Disease progression includes visual-field loss, deterioration of central vision, and structural decline, including narrowing of the ellipsoid zone and contraction of fundus-autofluorescence metrics [2,4]. Visual-field loss frequently drives blindness, while central visual acuity commonly deteriorates to severe levels by the fifth decade [1,3,5]. Recent real-world data also show substantial burden, including low-luminance difficulties, impaired mobility, depression, unemployment, and productivity loss [6].

Subretinal adeno-associated viral (AAV) RPGR gene augmentation has emerged as a major interventional strategy for RPGR-XLRP. Early human studies established the feasibility of subretinal RPGR gene delivery and suggested localized functional improvement in some treated participants [7,8]. Since then, the clinical landscape has expanded across multiple subretinal AAV-RPGR programs, including cotoretigene toliparvovec (AAV8-coRPGR), botaretigene sparoparvovec (AAV5-hRKp.RPGR), and AGTC-501 (rAAV2tYF-GRK1-RPGR), each differing in vector platform, promoter and transgene design, dose-escalation strategy, and treatment location within the retina [9,10]. Contemporary retinal gene-therapy development has shown that vector platform, route of delivery, first-in-human dose selection, immunogenicity, and trial design can substantially influence both safety and the interpretation of efficacy; related AAV products therefore should not be treated as biologically equivalent interventions in pooled analyses [11,12]. The marked interocular symmetry reported in RPGR-XLRP further illustrates both the opportunities and the methodological complexity of interventional trial design in this disease [13].

Functional endpoint selection remains challenging in RPGR-XLRP. Best-corrected visual acuity (BCVA) may remain relatively preserved until later disease stages because foveal function can persist despite ongoing centripetal degeneration, limiting its sensitivity to early progression or localized treatment effects [4,14,15,16]. Microperimetry provides fundus-tracked assessment of retinal sensitivity, whereas low-luminance visual acuity (LLVA) may capture central dysfunction not reflected by standard acuity; both have therefore been used to characterize functional change after treatment [8,14,16]. However, retinal-sensitivity estimates may vary with instrument, luminance condition, and test grid, and pointwise measures may be less stable than global metrics [15,17,18]. In the 24-month XOLARIS natural-history study, functional changes were generally small despite measurable anatomical progression, illustrating the difficulty of detecting short-term change in this disease [19,20].

The safety profile of subretinal AAV-RPGR therapy is also difficult to summarize as a single signal. Across early clinical programs, reported ocular adverse events have included corticosteroid-responsive intraocular inflammation, surgery- or procedure-related complications, and retinal pigment epithelium changes at higher exposure levels [7,9,10]. These observations suggest that ocular adverse events in RPGR gene therapy should not be treated as one homogeneous category, but rather as a mixture of procedure-related effects, inflammatory responses, and dose- or platform-context biological toxicity.

A recent systematic review and meta-analysis [21] provided the first quantitative synthesis of this literature; several methodological and interpretive uncertainties nevertheless remained regarding cohort dependence, paired-eye comparisons, comparator design, endpoint compatibility, and program-specific safety context. The evidence base is still shaped by small sample sizes, predominantly early-phase designs, heterogeneous endpoint definitions and dose reporting, variable follow-up durations, and nonuniform safety reporting. In this setting, the main unresolved clinical question is not only whether pooled estimates favor treatment, but how much confidence can reasonably be assigned to the apparent functional and ocular-safety signals before they guide clinical interpretation, within-program dose selection, delivery approach, endpoint choice, or future trial design [14,15,16,17,18,20].

We therefore performed a systematic review and meta-analysis of subretinal AAV-RPGR gene therapy in RPGR-associated X-linked retinitis pigmentosa, with emphasis on functional outcomes and clinically interpretable ocular safety. Consistent with our registered exploratory objective to identify knowledge gaps and support future clinical development and regulatory assessment, we used a dependence-aware analytic approach that restricted quantitative pooling to compatible measurement frameworks, incorporated observed paired-eye contrasts when available, evaluated residual within-participant dependence through sensitivity analyses, and preserved program-specific dose and delivery context. The aim was to characterize the direction, uncertainty, and clinical context of the observed functional and ocular-safety findings. Statistical non-confirmation was interpreted as a limit on the certainty supported by the current evidence rather than as proof of absent biological or clinical activity. This review is intended to support clinical interpretation and the design of future confirmatory trials without assigning greater certainty than the data allow.

## 2. Materials and Methods

### 2.1. Protocol Registration and Amendments

This systematic review and meta-analysis was registered in the International Prospective Register of Systematic Reviews (PROSPERO; CRD420251163589) and is reported in accordance with the Preferred Reporting Items for Systematic Reviews and Meta-Analyses (PRISMA) 2020 statement [22] (Table S5). The review commenced on 1 October 2025. The initial formal search was completed and archived on 8 October 2025; the PROSPERO record was submitted on 8 October 2025 and registered on 9 October 2025. At registration, screening, data extraction, risk-of-bias assessment, and quantitative synthesis had not begun.

The contemporaneous PROSPERO version 1.0 record was retained as the original registered plan. Analyses otherwise followed the registered protocol; material post-registration amendments, and implementation decisions, including their timing, rationale, and effect on eligibility, synthesis, and interpretation, are documented in the supplementary amendment log (Table S13).

### 2.2. Eligibility Criteria

Eligible studies were interventional clinical trials (phase 1–3) evaluating subretinal AAV-mediated RPGR gene augmentation in patients with genetically confirmed RPGR-associated X-linked retinitis pigmentosa, reporting at least one quantitative efficacy or safety outcome. Both randomized and non-randomized open-label or dose-escalation designs were eligible, as were single-arm studies using the untreated fellow eye or baseline as an internal comparator. Studies of other inherited retinal diseases, non-gene-based interventions, non-subretinal delivery, and preclinical models were excluded, as were reviews, meta-analyses, conference abstracts, and case series.

Structured trial-registry results with sufficient study-level information were eligible for narrative synthesis when no peer-reviewed full report was available. Quantitative inclusion additionally required an outcome estimate compatible with the prespecified estimand, scale, unit of analysis, and timepoint.

### 2.3. Information Sources, Search, and Selection

PubMed/MEDLINE, Web of Science, Embase, Scopus, and ClinicalTrials.gov were searched without date restrictions. The searches were initially conducted for the registered review and updated in July 2026; exact platform-specific run dates, complete strategies, and source-level record counts are provided in Table S1. Additional sources included the Cochrane Central Register of Controlled Trials (CENTRAL), the World Health Organization International Clinical Trials Registry Platform (WHO ICTRP), and backward and forward citation searching. Records imported into Rayyan (web application; https://www.rayyan.ai/) were screened independently by two reviewers (C.R.M.-d.-O.-S. and J.A.G.-C.), with disagreements resolved by discussion. CENTRAL and WHO ICTRP records were reviewed outside Rayyan as supplementary checks and were not included in the Rayyan deduplication counts. Structured registry-results reports identified through the platform searches were assessed separately for eligibility for narrative or quantitative synthesis. Full search strategies and source-level record counts are provided in Table S1.

### 2.4. Data Extraction and Administered-Dose Documentation

Data were extracted independently in duplicate by two reviewers (C.R.M.-d.-O.-S. and J.A.G.-C.). When numerical values were available in the manuscript text, tables, figure labels, or footnotes, these reported values were prioritized. WebPlotDigitizer (web application; https://automeris.io/WebPlotDigitizer/) was used only when outcome data were available exclusively in graphical form; extracted values were checked for consistency with the corresponding figure scale, axis labels, legends, and any related numerical information reported in the publication or Supplementary Material.

Administered dose was documented on a common per-eye basis by expressing the reported dose per treated eye while preserving the original unit terminology (vg or gp). When dose was reported per eye, the published value was retained; when concentration was reported, the administered amount per eye was calculated as reported concentration multiplied by injected volume. Source-reported units, injection volumes, per-eye calculation methods, source locations, and analytic handling are summarized in Table S10.

Because administered dose is inseparable from vector platform, capsid, promoter, transgene construct, titration method, injected volume, and transduction efficiency, doses were interpreted descriptively within each clinical program. Source-reported dose arms remained visible, and the HORIZON above-MTD cohort was displayed separately and excluded from the primary efficacy pool because it exceeded that program’s maximum tolerated dose. No cross-platform dose stratification, equipotency, or dose–response relationship was inferred.

### 2.5. Outcomes

The pre-specified primary outcomes were the change from baseline in mean retinal sensitivity by microperimetry at approximately six months and ocular safety, assessed as the frequencies of ocular serious adverse events and overall ocular adverse events during follow-up. To preserve measurement-scale comparability, the primary microperimetry synthesis was restricted to mesopic Macular Integrity Assessment (MAIA; CenterVue S.p.A., Padova, Italy) 68-loci acquisitions; scotopic static perimetry and peripheral-injection cohorts were excluded from this pool. For continuous functional outcomes, the estimand was the between-condition difference in change from baseline. When participant-level bilateral data were available, treated-minus-fellow-eye changes and their empirical paired sampling variance were calculated directly. Overall ocular adverse-event terms were pooled only when participant-level deduplication was possible; otherwise, clinically interpretable safety categories were synthesized when derivable and the remaining terms were reported descriptively. Cohorts without a derivable participant-level composite were excluded from the corresponding pooled proportion; clinically interpretable categories were synthesized when available, and remaining terms were reported descriptively.

Secondary outcomes were BCVA, low-luminance visual acuity (responder analysis), and the microperimetry responder rate, defined as the proportion of eyes meeting a study-defined retinal-sensitivity responder criterion, each at the timepoints at which comparable data were available. Microperimetry responder outcomes were considered eligible for quantitative synthesis only when responder definitions were compatible in perimetric modality, tested loci, and confirmation requirements across visits. For pooled retinal-sensitivity analyses, we prioritized the continuous mean difference because it preserved the original MAIA decibel scale and avoided dichotomizing the same underlying measurements [15,17].

### 2.6. Statistical Analysis

Analyses were performed in Python 3.12.3 using NumPy 2.4.4 and SciPy 1.17.1 [23,24]; figures were generated with Matplotlib 3.10.8. The inferential approach was selected according to the number of independent analytic cohorts and the outcome type. Primary continuous-outcome syntheses used one effect per independent analytic cohort: three cohorts for mesopic microperimetry at six months, two cohorts at twelve months, and three cohorts for BCVA at six months. Random-effects models used DerSimonian–Laird between-cohort variance (τ^2^) and conventional Hartung–Knapp–Sidik–Jonkman (HKSJ) small-sample confidence intervals without ad hoc variance truncation [25,26]. Conventional DerSimonian–Laird normal-approximation intervals were reported as sensitivity analyses. The twelve-month microperimetry synthesis was considered exploratory because only two independent cohorts contributed (df = 1).

For the dichotomous low-luminance responder outcome, in which only two studies contributed and HKSJ inference is uninformative (1 degree of freedom), a Mantel–Haenszel fixed-effect risk ratio was used as the primary effect measure. Odds-ratio and random-effects estimates were retained as sensitivity measures. The risk ratio was selected because responder proportions were not rare, making the odds ratio more likely to appear numerically farther from the null. Because each functional outcome was restricted to a single measurement instrument and scale (mesopic MAIA 68-loci microperimetry in decibels; ETDRS letters for BCVA), continuous outcomes were synthesized as raw mean differences with 95% confidence intervals rather than as standardized mean differences. Low-luminance visual-acuity responder outcomes were synthesized as Mantel–Haenszel risk ratios without continuity correction, as no zero-event cells were present. Analyses with only two contributing cohorts and sparse event counts were designated exploratory.

Dependence was addressed before pooling. Arm-level contrasts were retained for descriptive presentation. For XIRIUS Part 1, participant-level bilateral data were used to calculate observed treated-minus-fellow-eye changes and their empirical paired variance for microperimetry and BCVA. For XIRIUS Part 2, the two treated arms were combined and the full randomized untreated group was included once, consistent with Cochrane Handbook guidance for multi-arm trials [27]. For HORIZON microperimetry, cohort-level treated–fellow-eye estimates were retained, and their sampling variance was examined over assumed within-participant correlations ranging from 0 to 0.75 because the correlation was unreported. The botaretigene estimate was treated as one mixed-design cohort-level comparison. Multiple reports of the same participant cohort were linked and contributed only once to each outcome and timepoint. Comparator structures and the exclusion of external natural-history controls are summarized in Table S11.

Heterogeneity was assessed using I^2^, with τ^2^ estimated as part of the random-effects models. For the principal safety outcomes, the primary estimand was the aggregate event proportion among centrally injected treated eyes, with exact Clopper–Pearson 95% confidence intervals. An all-groups analysis that also included peripheral-injection cohorts was performed as a sensitivity analysis. Two-stage random-effects logit models with HKSJ intervals were retained as sensitivity analyses; for retinal detachment, a conventional Wald-z interval was additionally reported in Table S6, Panel B, alongside beta-binomial and one-stage binomial-normal models. Because fewer than ten studies contributed to any outcome, small-study effects were not formally assessed with funnel plots.

Additional sensitivity analyses included descriptive arm-level pooling, leave-one-cohort-out analyses, a reliability analysis excluding the XIRIUS Part 1 microperimetry acquisition described as unreliable, and assumed HORIZON fellow-eye correlations of ρ = 0, 0.25, 0.50, and 0.75 (Table S7). Robustness was further examined using REML and Paule–Mandel heterogeneity estimators, maximum-likelihood profile-likelihood intervals, and 95% prediction intervals based on t_{k − 2}; prediction intervals were not estimated when k = 2 (Table S8).

### 2.7. Risk of Bias and Certainty of Evidence

Risk of bias was assessed per outcome, with the tool matched to the design of the result contributed by each study. The randomized XIRIUS Part 2 comparison [28] was assessed with the Cochrane RoB 2 tool [29]. Non-randomized or mixed-design efficacy comparisons were assessed with ROBINS-I [30]: XIRIUS Part 1, assessed as a single study from its two reports [7,8]; HORIZON [10]; and the pooled dose-escalation and dose-expansion efficacy comparison of Michaelides et al. [9]. Because the botaretigene efficacy estimate used in the meta-analysis combines randomized expansion participants with non-randomized escalation participants against a pre-treatment deferred control, ROBINS-I rather than RoB 2 was applied to that result. Single-arm adverse-event proportions lack a comparator and do not estimate comparative causal effects; they were therefore assessed using a review-specific five-domain framework covering participant selection, event ascertainment, outcome definition, completeness of reporting, and selective reporting. Two reviewers (C.R.M.-d.-O.-S. and N.N.) completed each risk-of-bias assessment independently, resolving disagreements by discussion. Tool-selection rationale and domain-level judgments are provided in Table S2.

Certainty of evidence was rated per outcome using the Grading of Recommendations Assessment, Development and Evaluation (GRADE) approach [31]. Using the ROBINS-I-integrated GRADE approach, the body of evidence for each outcome began at high certainty and was rated downward according to the risk-of-bias judgment together with inconsistency, indirectness, imprecision, and publication bias [32]. Results are summarized in a summary-of-findings table, with the full evidence profile and the reason for each rating decision given in Table S3. Although the protocol specified GRADEpro GDT, certainty ratings were implemented manually to accommodate outcome-level integration of RoB 2, ROBINS-I, and the review-specific non-comparative safety framework [32].

### 2.8. Deviations from the Registered Protocol

The protocol prespecified the review population, interventions, comparators, eligibility criteria, outcome domains, random-effects synthesis, and GRADE assessment. Post-registration implementation decisions concerned multi-arm and within-participant dependence, restriction of microperimetry pooling to a common measurement framework, assessment of non-comparative harms, sparse-event sensitivity models, and non-performance of planned responder and subgroup syntheses. Their timing, rationale, and effect on eligibility, synthesis, and interpretation are documented in the amendment log (Table S13); none altered the review population, intervention, comparator, or core outcome domains.

## 3. Results

### 3.1. Study Selection and Characteristics

**Figure 1:**
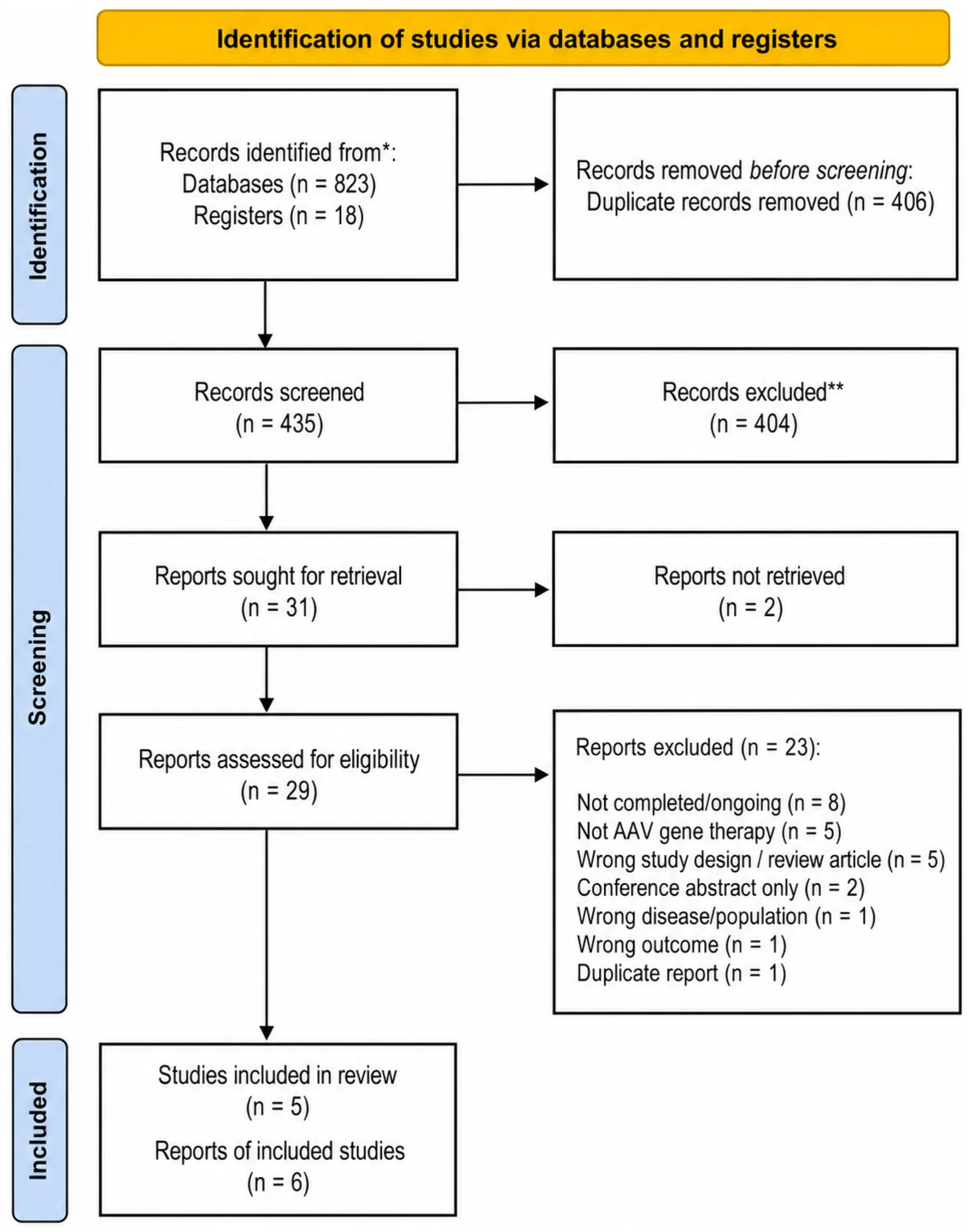
Preferred Reporting Items for Systematic Reviews and Meta-Analyses (PRISMA) 2020 flow diagram of study identification, screening, eligibility assessment, and inclusion. * Database records comprised PubMed/MEDLINE (*n* = 123), Web of Science Core Collection (*n* = 265), Embase (*n* = 263), and Scopus (*n* = 172); register records comprised ClinicalTrials.gov (*n* = 18). CENTRAL (*n* = 13) and WHO ICTRP (47 records representing 26 trials) were searched as supplementary sources and were not included in the primary deduplication counts. ** Records excluded during title and abstract screening did not meet the predefined eligibility criteria. Five studies reported in six records were included in the systematic review; four studies reported in five records contributed to the quantitative meta-analysis, whereas one structured registry-results report, identified through the updated WHO ICTRP search outside the primary deduplication workflow, was included in the narrative synthesis only. Adapted from the PRISMA 2020 flow diagram [22].

The flow of study identification, screening, eligibility assessment, and inclusion is shown in Figure 1. Five studies reported in six records met the inclusion criteria (Table 1). Four studies reported in five peer-reviewed publications contributed to the quantitative synthesis, comprising 128 enrolled or randomized participants and a treated-eye analytic set of 102 eyes across three AAV serotypes: the AAV8 cotoretigene toliparvovec program (XIRIUS Part 1, reported by Cehajic-Kapetanovic et al. [7] and, for the same cohort at later timepoints, von Krusenstiern et al. [8]; and XIRIUS Part 2, Lam et al. [28]); the AAV5-hRKp.RPGR botaretigene sparoparvovec program [9]; and the rAAV2tYF-GRK1-RPGR program AGTC-501 (HORIZON; Yang et al. [10]). Across the quantitatively contributing reports, follow-up ranged from 6 to 24 months. Cehajic-Kapetanovic et al. [7] and von Krusenstiern et al. [8] report the same XIRIUS Part 1 cohort; the later report was therefore not pooled separately for the continuous microperimetry or BCVA syntheses. Its nonoverlapping per-arm low-luminance responder counts contributed to the analysis shown in Figure S1.

The updated registry search additionally identified the phase 3 LUMEOS trial (NCT04671433/MGT-RPGR-021) [33], available as a structured registry-results report, but not yet as a peer-reviewed full article. LUMEOS evaluated bilateral botaretigene treatment and reported 52-week binocular vision-guided mobility and static-perimetry outcomes. Because its endpoint framework, bilateral design, and reporting format were not compatible with the predefined quantitative syntheses, the trial was retained for narrative synthesis only and contributed no participant or eye to any pooled estimate or quantitative-analysis denominator. Detailed participant, cohort, outcome, and denominator mapping is provided in Table S4.

### 3.2. Mean Retinal Sensitivity

**Figure 2:**
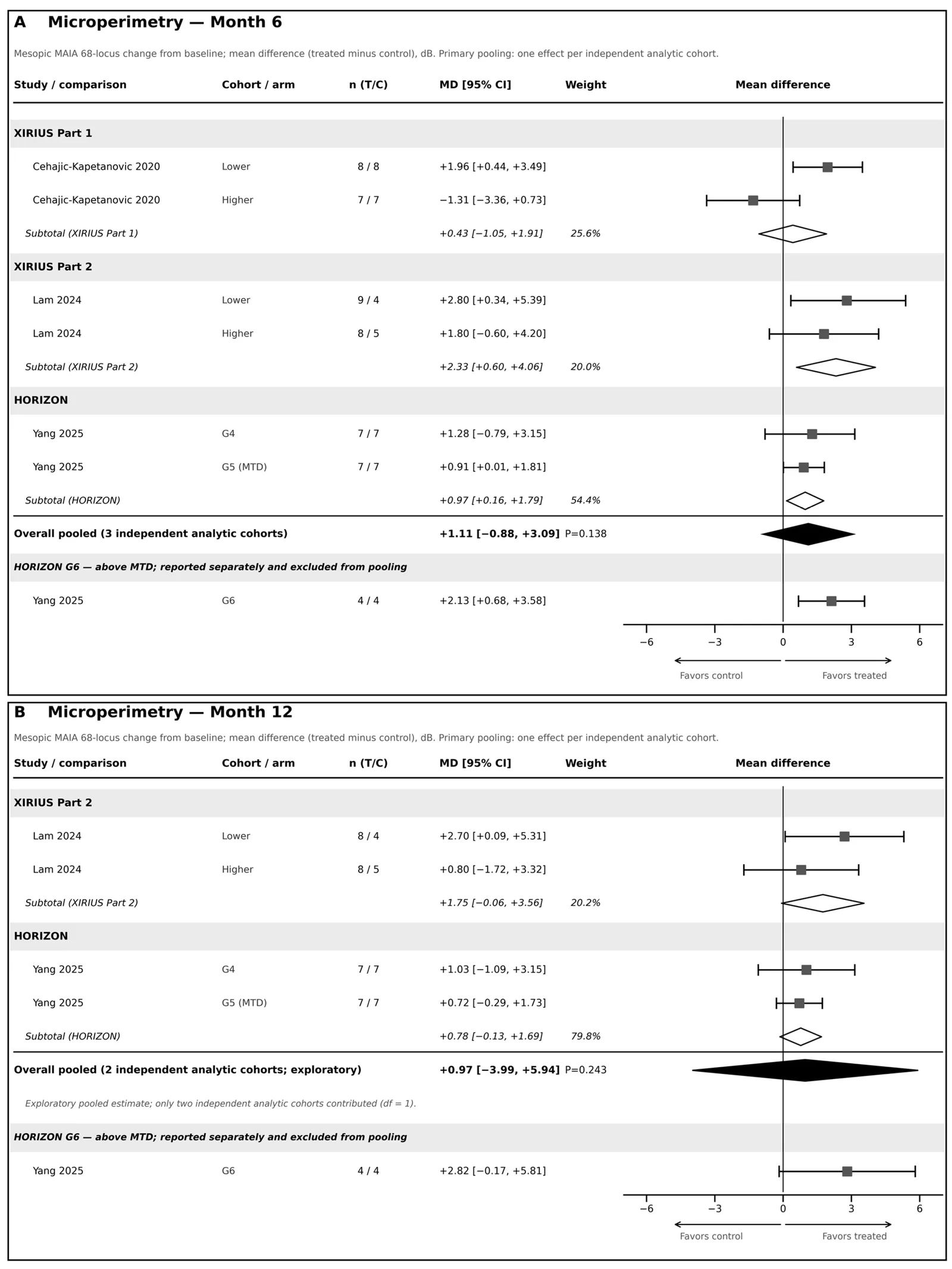
Microperimetry outcomes at Months 6 and 12. Mean differences in change from baseline in mesopic MAIA 68-locus retinal sensitivity between treated and untreated eyes or groups. Arm-level estimates are shown descriptively with equal-sized squares; open diamonds represent the independent analytic-cohort estimates used in pooling; filled diamonds represent the overall estimates. HORIZON G6 exceeded that program’s maximum tolerated dose and was excluded from pooling. The Month-12 synthesis was exploratory because only two cohorts contributed. Abbreviations: T/C, treated/control or treated/fellow eye; MAIA, Macular Integrity Assessment; MTD, maximum tolerated dose. Source studies: Cehajic-Kapetanovic et al. [7], Lam et al. [28], and Yang et al. [10].

At six months, treated eyes or groups showed a modest positive difference in mean retinal sensitivity relative to their contemporaneous untreated comparators, with a pooled mean difference of +1.11 dB across three independent analytic cohorts (Figure 2A). Under the primary HKSJ model, this estimate was not statistically significant (95% CI, −0.88 to +3.09; *p* = 0.138; I^2^ = 28%; τ^2^ = 0.18). Arm-level estimates are displayed descriptively, whereas XIRIUS Part 1, XIRIUS Part 2, and HORIZON each contributed one cohort-level estimate to the primary synthesis. The conventional DerSimonian–Laird normal-approximation sensitivity analysis was narrower and nominally significant (95% CI, +0.25 to +1.96; *p* = 0.011), but this result was considered model dependent and non-confirmatory.

At twelve months, the pooled mean difference was +0.97 dB across two independent analytic cohorts (Figure 2B), with no observed heterogeneity (I^2^ = 0%). Because only two cohorts contributed, this synthesis was exploratory; the primary HKSJ interval was wide and crossed the null (95% CI, −3.99 to +5.94; *p* = 0.243). The conventional DerSimonian–Laird normal-approximation sensitivity analysis was again narrower and nominally significant (95% CI, +0.16 to +1.79; *p* = 0.019), but was not interpreted as confirmatory. HORIZON Group 6, which exceeded that program’s maximum tolerated dose, was displayed separately and excluded from the pooled efficacy estimates; its descriptive mean differences were +2.13 dB (95% CI, +0.68 to +3.58) at six months and +2.82 dB (95% CI, −0.17 to +5.81) at twelve months. Overall, the pooled mesopic estimate remained approximately +1 dB at both timepoints, but no primary HKSJ analysis statistically confirmed a treatment effect.

Separately, the phase 3 LUMEOS report [33] showed a week-52 least-squares mean difference versus control of +1.24 dB (95% CI, +0.54 to +1.94) for central-field static-perimetry mean retinal sensitivity and +0.92 dB (95% CI, +0.29 to +1.55) for full-field mean retinal sensitivity. These static-perimetry outcomes were retained for narrative synthesis only and were not entered into the mesopic MAIA pool.

Dependence was addressed before pooling by using one effect per independent analytic cohort and, for XIRIUS Part 1, calculating the cohort contribution directly from observed treated-minus-fellow-eye changes and their empirical paired variance. For HORIZON, varying the assumed fellow-eye correlation from 0 to 0.75 changed the six-month pooled estimate only from +1.11 to +1.07 dB and the twelve-month estimate from +0.97 to +0.85 dB; all eight correlation-specific HKSJ analyses remained nonsignificant. Excluding the single XIRIUS Part 1 acquisition described as unreliable produced a six-month estimate of +1.13 dB (95% CI, −0.77 to +3.03; *p* = 0.124), without changing the interpretation (Table S7). Alternative Paule–Mandel and REML heterogeneity estimators yielded similar six-month point estimates and HKSJ intervals that crossed the null. Profile-likelihood and conventional normal-approximation sensitivities were narrower, but the six-month prediction interval remained wide (−6.61 to +8.82 dB), indicating substantial uncertainty in the effect expected in a future comparable cohort (Table S8).

### 3.3. Microperimetry Responder Rate

The planned microperimetry responder synthesis was not performed because the responder definitions were not analytically comparable across trials (Table S12); study-level findings were therefore summarized descriptively. In XIRIUS Part 1 (von Krusenstiern et al. [8]; AAV8; ≥7 dB at ≥5 of 16 central loci, single visit), 6 of 12 treated eyes (50%) in cohorts 3–6 met the criterion at one month, and 4 of 12 (33%) remained responders at both six and twelve months, versus 1 of 12 untreated fellow eyes (8%) at twelve months. In XIRIUS Part 2 (Lam et al. [28]; AAV8; the same 16-central-loci definition), the twelve-month primary endpoint was not met: three of eight low-dose eyes (37.5%), two of eight high-dose eyes (25.0%), and two of nine untreated control eyes (22.2%) were responders.

In HORIZON (Yang et al. [10]; rAAV2tYF-GRK1-RPGR; a stricter definition of ≥7 dB at ≥5 of all 68 loci confirmed on two consecutive visits), two of seven Group 4 participants were responders at both six and twelve months; in Group 5, three of seven were responders at six months and four of seven at twelve months. No untreated fellow eye met the criterion at either timepoint. The above-MTD cohort (Group 6) had two of four responders at both six and twelve months and was reported separately. Botaretigene sparoparvovec (Michaelides et al. [9]; AAV5) did not report a mesopic-microperimetry responder rate, assessing responders instead by static photopic and scotopic perimetry, and therefore could not contribute to this outcome. LUMEOS [33] likewise reported static-perimetry pointwise-response outcomes rather than a mesopic MAIA responder definition and was retained as narrative evidence only. Findings generally favored treated eyes, but the criteria were not interchangeable; comparable responder definitions came from a single program, and event counts were small; no pooled estimate was computed.

### 3.4. Best-Corrected Visual Acuity

**Figure 3:**
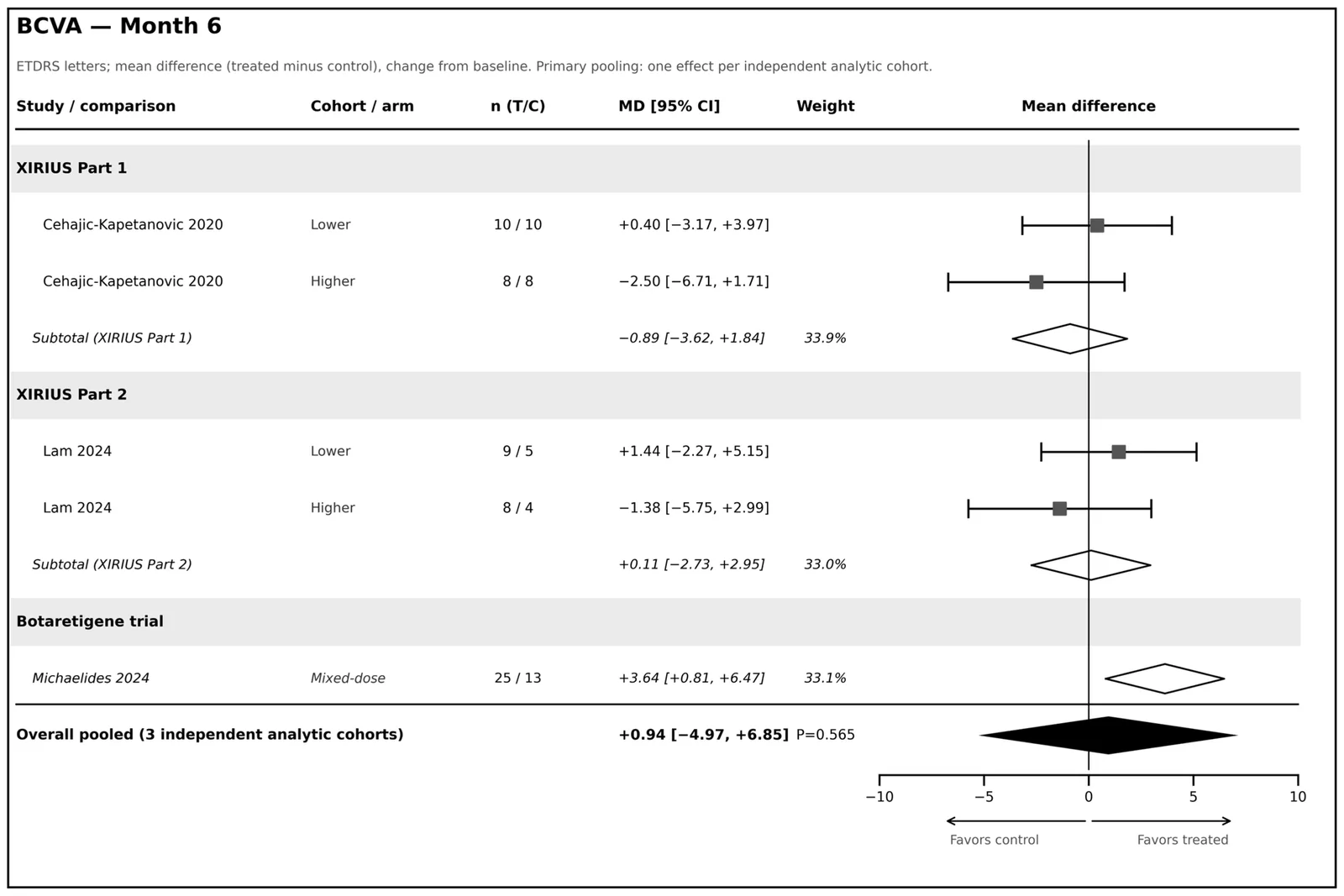
Best-corrected visual acuity at Month 6. Mean differences in change from baseline between treated and untreated eyes or groups, expressed in ETDRS letters. Arm-level estimates are shown descriptively with equal-sized squares; open diamonds represent the independent analytic-cohort estimates used in pooling; the filled diamond represents the overall estimate. The botaretigene estimate represents the mixed-design comparison against the deferred-treatment control. Abbreviations: T/C, treated/control or treated/fellow eye; ETDRS, Early Treatment Diabetic Retinopathy Study. Source studies: Cehajic-Kapetanovic et al. [7], Lam et al. [28], and Michaelides et al. [9].

At six months, three independent analytic cohorts contributed to the BCVA synthesis. The pooled mean difference was +0.94 ETDRS letters (Figure 3), with no statistically significant difference under the primary HKSJ inference (95% CI, −4.97 to +6.85; *p* = 0.565; I^2^ = 64%; τ^2^ = 3.62 letters^2^). The conventional DerSimonian–Laird normal-approximation sensitivity analysis was narrower, but also nonsignificant (95% CI, −1.75 to +3.63; *p* = 0.494). The five arm-level contrasts are displayed descriptively, whereas XIRIUS Part 1, XIRIUS Part 2, and the botaretigene trial each contributed one cohort-level estimate to the primary pool. The XIRIUS Part 1 contribution was calculated directly from observed participant-level treated–fellow-eye changes and did not require an assumed correlation. Leave-one-cohort-out and alternative heterogeneity-estimator analyses did not change the interpretation, and the prediction interval was wide (−28.87 to +30.75 letters; Tables S7 and S8).

At twelve months, XIRIUS Part 2 was the only peer-reviewed study reporting continuous BCVA against a concurrent untreated control, and no benefit was observed. LUMEOS [33] showed a week-52 least-squares mean difference versus the control of +0.96 ETDRS letters (95% CI, −0.84 to +2.76), which was retained for narrative synthesis only. At twenty-four months, only Yang et al. [10] reported BCVA, with a non-poolable single-study change in centrally treated eyes (+3.1 letters, SD 8.6) versus fellow-eye controls (+0.3 letters, SD 3.47). No pooled or narratively reported BCVA comparison demonstrated a confirmed benefit.

### 3.5. Low-Luminance Visual Acuity

As a secondary outcome, the proportion of eyes achieving a ≥10-ETDRS-letter low-luminance acuity gain was compared between treated and untreated eyes, pooling the two treatment arms of Lam et al. [28] against their untreated control together with the dosed and untreated fellow eyes of von Krusenstiern et al. [8]. At six months, the response was more frequent in treated than in control eyes (11/28, 39%, versus 2/20, 10%), but the pooled Mantel–Haenszel risk ratio did not reach statistical significance (RR 3.83; 95% CI, 0.94 to 15.57; Figure S1A). The difference attenuated by twelve months (treated 9/28, 32%, versus control 5/20, 25%; RR 1.46; 95% CI, 0.58 to 3.71; Figure S1B). For XIRIUS Part 1, the published reports provided marginal treated-eye and fellow-eye responder counts, but not the discordant-pair counts required to reconstruct an exact matched-eye binary analysis. Cohort- and arm-level event counts are shown in Figure S1.

LUMEOS [33] evaluated continuous monocular LLVA rather than the ≥10-letter responder outcome and reported a week-52 least-squares mean difference versus control of +4.75 ETDRS letters (95% CI, +1.64 to +7.86); this result was retained for narrative synthesis only and was not combined with the responder analysis. A more stringent ≥15-letter low-luminance gain was reported in a single trial only and was not quantitatively synthesized. Given the sparse responder-event counts, wide confidence intervals, and sensitivity of the six-month estimate to a single control event, the pooled ≥10-letter result remains exploratory and hypothesis generating rather than evidence of confirmed efficacy.

### 3.6. Safety

**Figure 4:**
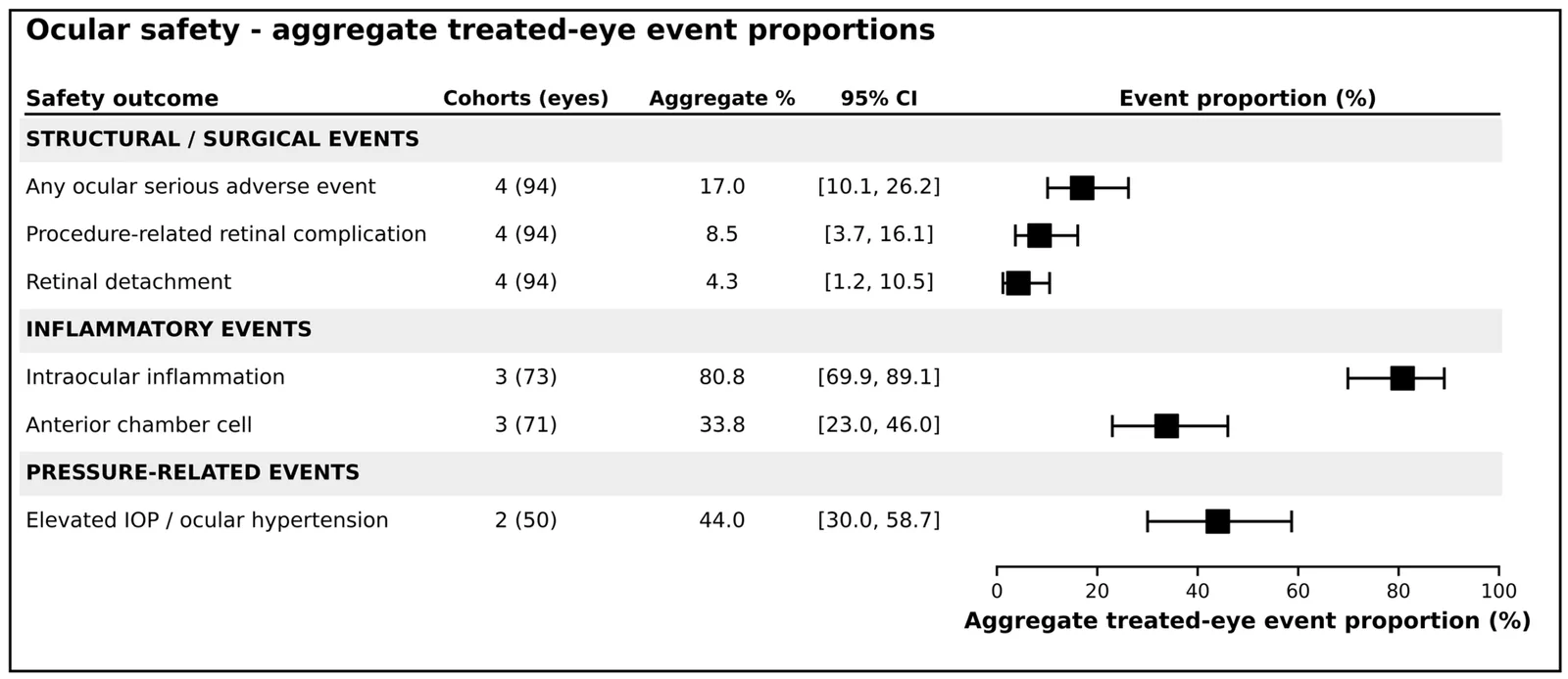
Ocular safety event proportions in central-injection cohorts. Aggregate treated-eye proportions with exact Clopper–Pearson 95% confidence intervals. Squares represent point estimates, and horizontal lines represent the corresponding 95% confidence intervals. Intraocular inflammation is a deduplicated composite, whereas anterior chamber cell and elevated intraocular pressure/ocular hypertension are component outcomes. Denominators include only cohorts reporting explicit participant-level counts; nonreporting cohorts were excluded rather than treated as having zero events. Trial-level counts and model-based sensitivity analyses are provided in Tables S9 and S6, respectively.

Aggregate central-injection treated-eye event proportions were calculated from participant-level counts using exact Clopper–Pearson 95% confidence intervals as the primary estimates (Figure 4); random-effects and alternative sparse-event models were retained as sensitivity analyses (Table S6). Among 94 centrally injected treated eyes, any ocular serious adverse event occurred in 16 eyes (17.0%; 95% CI, 10.1–26.2), procedure-related retinal complications in 8 eyes (8.5%; 95% CI, 3.7–16.1), and retinal detachment in 4 eyes (4.3%; 95% CI, 1.2–10.5). Deduplicated intraocular inflammation was reported for three derivable cohorts and occurred in 59 of 73 treated eyes (80.8%; 95% CI, 69.9–89.1). HORIZON reported potentially overlapping inflammatory preferred terms without a derivable central-cohort composite and was therefore excluded from that denominator. Anterior chamber cell was reported in 24 of 71 eyes (33.8%; 95% CI, 23.0–46.0), and elevated intraocular pressure or ocular hypertension in 22 of 50 eyes (44.0%; 95% CI, 30.0–58.7); these were descriptive component outcomes restricted to cohorts reporting explicit participant-level counts.

In the all-groups sensitivity analysis, which additionally included the eight peripherally injected HORIZON eyes, any ocular serious adverse event occurred in 19 of 102 eyes (18.6%; 95% CI, 11.6–27.6) and retinal detachment in 7 of 102 eyes (6.9%; 95% CI, 2.8–13.6). The difference in retinal-detachment frequency was driven by HORIZON, in which 3 of 8 peripherally injected eyes and 1 of 21 centrally injected eyes experienced the event. Exact binomial, beta-binomial, two-stage random-effects logit, and one-stage binomial-normal generalized linear mixed-model analyses produced concordant point estimates; the beta-binomial dispersion and GLMM between-program variance were estimated at the boundary, reflecting the limited information available from four programs rather than evidence of homogeneity (Table S6, Panel B).

Other ocular events were summarized descriptively when a comparable participant-level category could not be derived across programs, including retinal pigment epithelium or bleb-related atrophy, subcapsular cataract, and cystoid macular edema. In the above-MTD cohort, retinal pigment epithelium depigmentation occurred in three of four centrally dosed participants, with one additional retinotomy-site change; the next-lower dose (6.8 × 10^11^ vg/eye) was the maximum tolerated dose within that program. The above-MTD toxicity was summarized descriptively and was not pooled as an event proportion. No suspected unexpected serious adverse reactions or endophthalmitis were reported. One ocular serious adverse event was attributed by the source investigators to natural disease progression rather than to treatment or the surgical procedure; excluding this event yielded 16.0% (95% CI, 9.2 to 25.0).

The LUMEOS report [33] presented bilateral safety at the participant level and was therefore not combined with the eye-level early-phase proportions. Among 62 immediately treated participants, 10 experienced at least one serious adverse event, including one retinal detachment; four participants had serious adverse events considered related to surgery and one had an event considered related to botaretigene. Thirty-two of sixty-two participants (51.6%) had at least one inflammation-related treatment-emergent adverse event summarized across both eyes. Trial-level counts underlying the pooled early-phase safety proportions are provided in Table S9.

### 3.7. Risk of Bias

**Figure 5:**
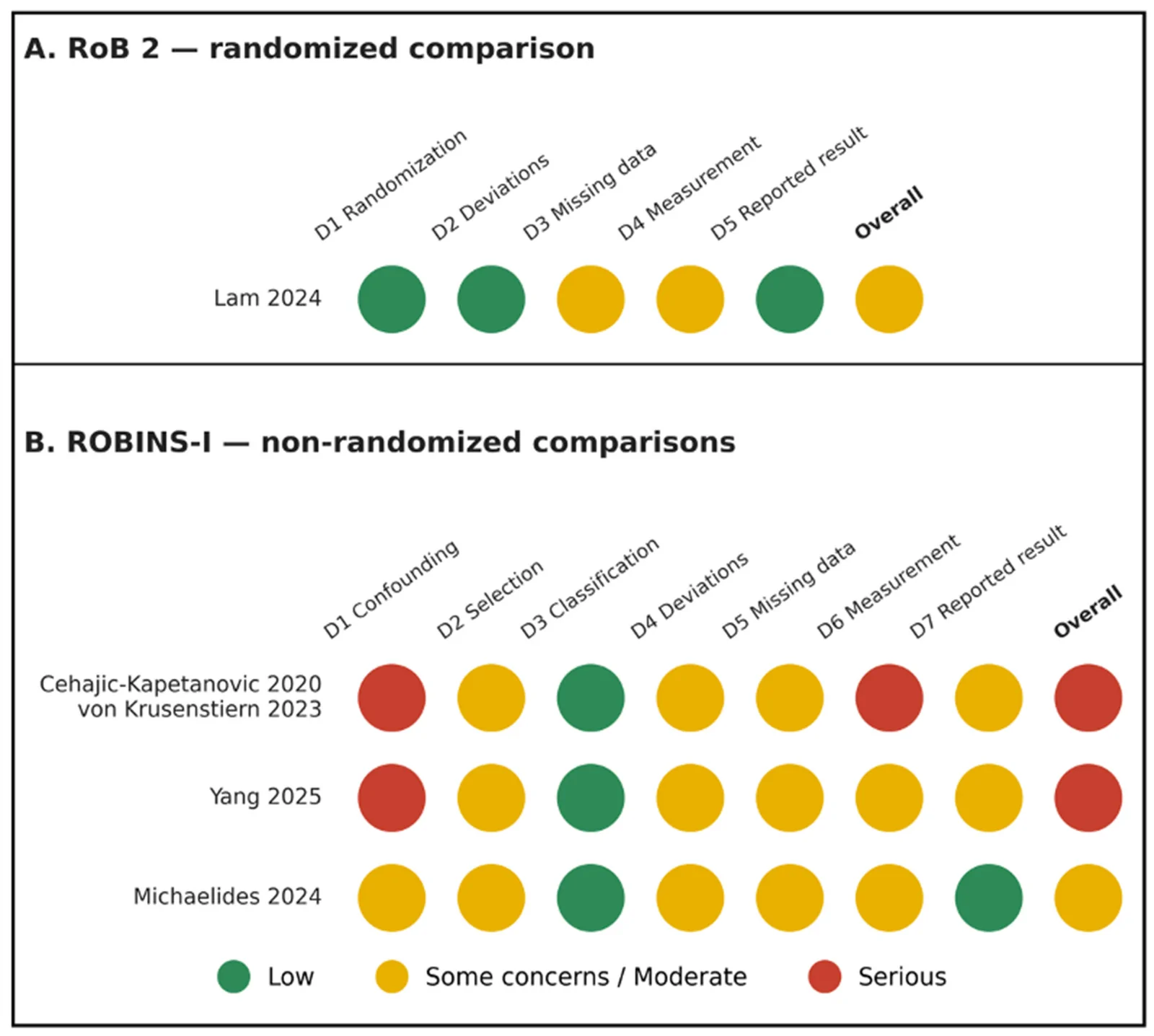
Risk-of-bias traffic-light plot for the functional efficacy outcomes. Panel (**A**) shows RoB 2 judgments for the randomized Lam 2024 comparison [28]; Panel (**B**) shows ROBINS-I judgments for the non-randomized and mixed-design comparisons. The Yang 2025 row represents the HORIZON comparison [10]. XIRIUS Part 1 is assessed as a single study from the Cehajic-Kapetanovic 2020 [7] and von Krusenstiern 2023 [8] reports. Michaelides 2024 [9] reflects the pooled dose-escalation and dose-expansion efficacy comparison and was assessed with ROBINS-I rather than RoB 2. Yellow denotes some concerns under RoB 2 and moderate risk of bias under ROBINS-I. Risk of bias was assessed per outcome; where domain judgments were identical across functional outcomes, they are displayed in a consolidated study-level row. Non-comparative safety outcomes were assessed separately using the review-specific five-domain framework and are reported in Table S2.

Risk of bias was assessed per outcome, using the tool matched to the design of the contributed result. The randomized XIRIUS Part 2 comparison [28] was judged as having some concerns overall for the functional outcomes, driven mainly by missing outcome data, including COVID-19-related attrition, and by the absence of participant masking for effort-dependent measurements. The non-randomized XIRIUS Part 1 [7,8] and HORIZON [10] comparisons were judged at serious overall risk of bias for the functional outcomes, primarily because of confounding inherent to their open-label dose-escalation designs and, for XIRIUS Part 1, open-label measurement of effort-dependent endpoints. The botaretigene efficacy comparison [9] was at moderate overall risk because its mixed design combined non-randomized escalation and randomized expansion participants against a deferred-treatment control. For the non-comparative safety proportions, intraocular inflammation, any ocular serious adverse event, and retinal detachment were each judged as having some concerns overall under the review-specific five-domain framework, principally because of investigator-led event classification for inflammation and incomplete intended follow-up in XIRIUS Part 2 and the botaretigene program. Small sample size, sparse events, and limited statistical power were considered under GRADE imprecision rather than risk of bias. Functional-outcome judgments are shown in Figure 5, and the complete functional and safety assessments are provided in Table S2.

### 3.8. Certainty of Evidence (GRADE)

Certainty was very low for mesopic retinal sensitivity at six and twelve months, six-month BCVA, and the six-month ≥10-letter low-luminance responder outcome; certainty was low for intraocular inflammation, any ocular serious adverse event, and retinal detachment (Table 2). No outcome retained moderate or high certainty. The functional outcomes were downgraded for risk of bias and very serious imprecision, reflecting the small number of independent analytic cohorts and confidence intervals that remained compatible with both no effect and clinically different effects. The twelve-month microperimetry estimate was particularly uncertain because only two independent cohorts contributed. Intraocular inflammation was downgraded for risk of bias and inconsistency in event definitions and observed proportions, whereas any ocular serious adverse event and retinal detachment were downgraded for risk of bias and imprecision. Indirectness was assessed separately rather than inferred solely from differences between vector platforms: the quantitative microperimetry synthesis was restricted to a compatible mesopic MAIA framework, and differences in vector, dose, delivery, and steroid regimen were retained in the clinical interpretation rather than treated as biologically equivalent. Publication bias could not be assessed formally because fewer than ten studies contributed to every outcome; this limitation was reported without assuming that publication bias was absent. The complete evidence profile and the rationale for every domain judgment are provided in Table S3.

## 4. Discussion

In this synthesis of subretinal AAV-RPGR gene therapy, mesopic retinal sensitivity favored treatment by +1.11 dB at six months and +0.97 dB at twelve months, but neither estimate reached statistical significance under the primary inference. No statistically significant pooled difference was observed for best-corrected visual acuity. The ≥10-letter low-luminance acuity responder outcome favored treatment at six months, but did not reach significance under the primary risk-ratio analysis; by twelve months, responder proportions were more similar between treated and control eyes (32% versus 25%). For safety, intraocular inflammation was frequent in the three cohorts with a derivable deduplicated composite, whereas structural or surgical complications were less frequent and varied by delivery route; the above-MTD HORIZON cohort showed distinct retinal pigment epithelium toxicity. Overall, the evidence does not establish a clinically actionable functional benefit, while the ocular safety findings represent uncontrolled treated-eye proportions that vary by program, delivery approach, and observation window.

The earlier synthesis [21] was the first quantitative summary of this early-phase literature and included largely the same studies and event data. Our results differ mainly because of how dependent comparisons, endpoint definitions, and study designs were handled. We used one estimate per independent cohort, counted shared controls once, pooled only mesopic MAIA 68-locus outcomes, and matched risk-of-bias tools to the design of each result. The prespecification status and timing of these decisions are described in Methods Section 2.8 and Table S13; the design-specific risk-of-bias and endpoint-compatibility assessments are detailed in Tables S2 and S12.

The direction of the findings was largely unchanged. For the six-month low-luminance responder outcome, the point estimate was nearly identical to the previous synthesis (RR 3.83 versus 3.79), but counting the shared Lam et al. control once widened the confidence interval to include the null. We did not pool microperimetry responder rates because definitions differed by modality, grid, and confirmation requirements; in a post hoc XIRIUS analysis [34], responder frequency ranged from 20% to 64% depending on the definition. Conventional normal-approximation intervals are reported alongside the primary analyses because the inferential approach materially affected interval width.

The approximately 1 dB differences at Months 6 and 12 should be interpreted as numerically similar, but statistically unconfirmed group-level signals, rather than as evidence that treatment had no effect. In RPGR-associated disease, post-familiarization coefficients of repeatability for MAIA mean sensitivity have ranged from approximately 0.7 to 1.3 dB [15,35], while broader retinitis pigmentosa data show greater intervisit variability in eyes with poorer visual acuity, reaching 2.4 dB [36]. Variability is greatest between the first and second examinations, and triplicate baseline testing has therefore been recommended in this population [35]. These individual-level repeatability limits are neither minimal clinically important differences nor direct measures of uncertainty for a pooled between-condition mean. Thus, an approximately 1 dB difference cannot be assumed to represent a reliably perceptible improvement in an individual patient, but its overlap with test–retest variability does not by itself establish that the group-level signal is measurement noise.

The clinical context supports retinal sensitivity as a relevant functional endpoint, but does not establish the patient-level importance of a 1 dB difference. In RPGR-associated retinopathy, low-luminance deficit correlated with whole-grid mean retinal sensitivity (ρ = −0.83), central four-locus sensitivity (ρ = −0.68), and ellipsoid-zone width (ρ = −0.76), supporting a relationship between microperimetric function and vision under reduced luminance [16]. More broadly in retinitis pigmentosa, mesopic and low-contrast testing detects functional impairment that standard high-contrast acuity may miss [37]. In XOLARIS, which used the same 68-locus metric, mean study-eye sensitivity declined by 0.7 dB in the lower-BCVA subgroup and 0.4 dB in the higher-BCVA subgroup over 24 months, while VFQ-25 composite scores declined by 2.8 and 0.6 points, respectively [19]. For scale only, the six-month pooled estimate was numerically larger than these small mean 24-month changes; however, this indirect comparison differs in estimand, timeframe, and population and does not establish clinical importance or a causal treatment effect.

Expected change also varies by phenotype and retinal region. Separate natural-history studies found a faster decline in central MAIA sensitivity in cone-rod than in rod-cone RPGR phenotypes [38] and faster static-perimetry loss near the ellipsoid-zone transition than within the macula [39]. These measures are not interchangeable with the 68-locus whole-grid mean, but they illustrate why a single numerical difference may not have the same meaning in every patient. Among the disease-specific studies reviewed, we identified no validated minimal clinically important difference for 68-locus mean retinal sensitivity in RPGR-associated XLRP. Given confidence intervals that crossed the null, the exploratory nature of the twelve-month estimate, and very-low-certainty evidence, the approximately 1 dB difference is best regarded as a modest, statistically unconfirmed group-level signal of uncertain patient-level relevance.

The dissociation between endpoints is also consistent with disease mechanism: because central acuity is relatively preserved until late in RPGR-related degeneration, BCVA is subject to a ceiling effect and is comparatively insensitive to localized treatment effects, whereas microperimetry and low-luminance acuity probe macular function that declines earlier and may better capture change [14,16,18]. The attenuation of the low-luminance responder difference by twelve months, together with the sparse event counts, precludes a firm mechanistic interpretation and underscores the need for endpoints that are both mechanistically aligned and analytically stable [15,20].

The LUMEOS structured registry-results report [33] provides additional, but not directly comparable, context. At week 52, model-adjusted differences favored botaretigene for static-perimetry mean retinal sensitivity and continuous LLVA, whereas BCVA did not demonstrate a significant difference and the primary binocular vision-guided mobility endpoint was not met. These least-squares mean differences are trial-model-adjusted estimates derived from bilateral treatment and different endpoint frameworks; they are therefore not interchangeable with the eye-level mean differences pooled for mesopic MAIA in this review. The findings suggest that treatment-related change may be detectable with selected functional measures, but their interpretation remains endpoint dependent. Because the results are currently available as a structured registry report rather than a peer-reviewed full article, they should be interpreted cautiously.

Publicly reported phase 3 evidence may materially change this synthesis. LUMEOS is the first randomized phase 3 trial in this indication with registry results; its peer-reviewed report and future confirmatory publications may provide larger samples, longer follow-up, and prospectively defined endpoints. Because such reports are likely to appear sequentially, a living systematic review with prespecified update triggers would be reasonable once new phase 3 data become available.

The safety findings should not be read as a single, uniform risk profile for AAV-RPGR gene therapy, because the observed events differ in their likely mechanism. Inflammatory events were frequent among the three central-injection cohorts with derivable participant-level composites (59/73 eyes; 80.8%) and may reflect vector or transgene exposure, with their observed frequency also influenced by nonuniform corticosteroid regimens and event definitions; anterior chamber cell was reported in 24/71 eyes (33.8%). Surgical or procedure-related complications, including retinal detachment and other bleb- or retinotomy-related events, occurred in 8/94 eyes (8.5%) and are most directly linked to subretinal delivery. Pressure-related events occurred in 22/50 eyes (44.0%) and may reflect inflammation, corticosteroid exposure, or both. Dose-related retinal toxicity was distinct: retinal pigment epithelium depigmentation occurred in three of four participants treated above the maximum tolerated dose in HORIZON [10], suggesting a program-specific tolerability ceiling rather than a class effect.

The clearest separation concerned delivery route. Including central and peripheral cohorts yielded 7 retinal detachments among 102 treated eyes (6.9%), whereas restriction to central injection yielded 4 among 94 (4.3%); 3 of the 4 HORIZON detachments occurred among only eight peripherally injected eyes. A comparable route-stratified assessment of inflammation was not possible because HORIZON reported overlapping inflammatory preferred terms without a deduplicated participant-level composite. Vector platform, administered dose, injection location, bleb configuration, surgical technique, corticosteroid regimen, and observation window differed between programs. These proportions therefore provide early program-dependent context rather than a transferable risk estimate for an individual AAV-RPGR product, and they represent cumulative treated-eye proportions over each program’s reported follow-up rather than exposure-adjusted incidence rates or interchangeable fixed-horizon risks [7,9,11]. Aggregate and model-based sensitivity analyses are reported in Table S6, with trial-level counts in Table S9.

These findings have several implications for future AAV-RPGR development. For dose selection, future development should remain within tolerated program-specific ranges rather than prioritize numerically larger descriptive effects observed at excessive exposure. For delivery approach, safety estimates should be interpreted and reported by injection route. For endpoint choice, the dissociation between a mechanistically aligned mesopic microperimetry difference and ceiling-limited BCVA, together with the instrument- and grid-dependence of responder detection, supports prospectively fixed, luminance- and grid-standardized functional endpoints. For trial design, the small number of independent cohorts, different comparator structures, and paired-eye dependence should inform endpoint selection, primary timepoint selection, and power calculations. Patient-centered endpoints should also be selected with recognition that responsiveness may differ substantially among microperimetry, low-luminance acuity, standard acuity, and mobility measures. These considerations provide a framework for interpreting the current evidence and for designing future trials that can more directly test the effects suggested by the available studies.

This review has important limitations. With only two or three independent cohorts per continuous outcome, small-sample random-effects inference can produce wide or unstable intervals [40]; alternative heterogeneity estimators, profile-likelihood analyses, and normal-approximation sensitivities did not overcome the underlying information limitation. Some inputs were derived from published figures by digitization, introducing extraction uncertainty that we mitigated by duplicate, cross-checked extraction. The natural-history comparison is indirect, and vector platform, administered dose, delivery approach, and clinical program are inseparable, so reliable cross-program subgroup inference was not feasible. The non-comparative safety assessment used a fully specified review-specific framework rather than a validated instrument, and the safety proportions remained affected by nonuniform definitions and observation periods. LUMEOS was available only through structured registry results and could contribute narratively but not to the quantitative syntheses. Consistent with these constraints, GRADE certainty was very low for all functional outcomes and low for the three principal safety outcomes. The estimates should therefore be interpreted as descriptive or hypothesis generating rather than as confirmation of clinical efficacy or comparative safety.

## 5. Conclusions

Current evidence suggests a small, statistically unconfirmed difference in mesopic retinal sensitivity favoring treatment. A modest effect cannot be excluded, but no clinically actionable functional benefit has been established. Safety findings include frequent intraocular inflammation, retinal detachment concentrated in the peripherally injected group, and retinal pigment epithelium toxicity at the above-MTD dose; these estimates are uncontrolled and program dependent. Given the very low certainty of the functional evidence, the observed functional signals remain hypothesis generating. Adequately powered randomized trials should use standardized, clinically interpretable functional endpoints, route-specific safety reporting, and within-program dose selection.

## Figures and Tables

**Table 1:** Characteristics of included subretinal AAV-RPGR gene therapy trials.

Study/Program	Vector Product	Design/ Comparator	Participants	Dose(s), as Reported	Outcomes/ Follow-Up
XIRIUS Part 1— Cehajic-Kapetanovic 2020 [7]; von Krusenstiern 2023 [8]	AAV8-coRPGR/cotoretigene	Phase 1/2 open- label dose escalation; untreated fellow eye	18 treated	Six escalating cohorts; 5 × 10^10^–5 × 10^12^ gp/mL	Microperimetry, BCVA, LLVA, safety; functional outcomes at 6–12 mo; safety through 24 mo
XIRIUS Part 2— Lam 2024 [28]	AAV8-coRPGR/cotoretigene	Phase 2/3 randomized 1:1:1; untreated control	32 randomized; 23 treated	5 × 10^10^, 2.5 × 10^11^ vg/eye	Microperimetry, BCVA, LLVA, safety; 12 mo
Botaretigene— Michaelides 2024 [9]	AAV5-hRKp.RPGR/botaretigene	Phase 1/2 escalation and expansion; deferred control	49 enrolled; 45 treated at some point (32 immediately treated; 13 deferred treated after week 26); 4 deferred discontinued before treatment	1.0, 2.0, 4.0 × 10^11^ vg/mL	BCVA, safety; 6–12 mo
HORIZON— Yang 2025 [10]	rAAV2tYF-GRK1-RPGR/AGTC-501	Phase 1/2 open- label dose escalation; untreated fellow eye	29 treated; 21 central, 8 peripheral	2.48 × 10^10^–1.99 × 10^12^ vg/eye	Microperimetry, BCVA, safety; 6–24 mo
LUMEOS—NCT04671433/MGT-RPGR-021	AAV5-hRKp.RPGR/ botaretigene	Phase 3 randomized controlled trial; bilateral immediate treatment versus deferred control	97 enrolled; 95 in the principal randomized population; 62 immediate treatment, 33 deferred control	2.0 × 10^11^ and 4.0 × 10^11^ vg/mL	Binocular vision-guided mobility and static-perimetry mean retinal sensitivity; LLVA, BCVA, and safety; 12 mo (week 52); narrative synthesis only

Doses are shown in the units reported by each trial and are interpreted only within each clinical program; no cross-platform dose stratification, equipotency, or dose–response relationship was inferred. Participant/outcome mapping and administered-dose documentation are provided in Tables S4 and S10. AAV, adeno-associated virus; BCVA, best-corrected visual acuity; gp, genome particles; LLVA, low-luminance visual acuity; MRS, mean retinal sensitivity; vg, vector genomes; RPGR, retinitis pigmentosa GTPase regulator; VMA, vision-guided mobility assessment.

**Table 2:** GRADE summary of findings.

Outcome (Timepoint)	Effect Estimate (95% CI)	Certainty	Main Reason(s) for DOWNGRADE
Mesopic retinal sensitivity, 6 mo	MD +1.11 dB (HKSJ −0.88 to +3.09)	⊕◯◯◯ Very low	Risk of bias; very serious imprecision
Mesopic retinal sensitivity, 12 mo	MD +0.97 dB (HKSJ −3.99 to +5.94)	⊕◯◯◯ Very low	Risk of bias; very serious imprecision
BCVA, 6 mo	MD +0.94 letters (HKSJ −4.97 to +6.85)	⊕◯◯◯ Very low	Risk of bias; very serious imprecision
Low-luminance VA gain ≥10 letters, 6 mo	RR 3.83 (0.94 to 15.57)	⊕◯◯◯ Very low	Risk of bias; very serious imprecision
Intraocular inflammation, central-injection treated-eye proportion	59/73; 80.8% (69.9 to 89.1)	⊕⊕◯◯ Low	Risk of bias in non-comparative safety evidence; inconsistency
Any ocular serious adverse event, central-injection treated-eye proportion	16/94; 17.0% (10.1 to 26.2)	⊕⊕◯◯ Low	Risk of bias in non-comparative safety evidence; imprecision
Retinal detachment, central-injection treated-eye proportion	4/94; 4.3% (1.2 to 10.5)	⊕⊕◯◯ Low	Risk of bias in non-comparative safety evidence; imprecision

Certainty was rated per outcome using the ROBINS-I-integrated GRADE approach [32]. Risk of bias was assessed with RoB 2 for randomized efficacy evidence, ROBINS-I for nonrandomized or mixed-design efficacy evidence, and a review-specific five-domain framework for non-comparative safety outcomes [29,30]. Functional outcomes used independent analytic cohort-level HKSJ inference; conventional z-based DL sensitivity analyses are reported in Table S7. Safety outcomes are presented as aggregate treated-eye proportions with exact Clopper–Pearson 95% confidence intervals; random-effects logit sensitivity analyses are reported in Table S6. CI, confidence interval; MD, mean difference; RR, risk ratio; BCVA, best-corrected visual acuity; SAE, serious adverse event; HKSJ, Hartung–Knapp–Sidik–Jonkman. Publication bias could not be assessed formally because fewer than ten studies contributed to any outcome. Single-arm proportions express absolute treated-eye risk without a comparator. Source studies are cited in the main text [7,8,9,10,28].

## Data Availability

All data analyzed in this systematic review and meta-analysis were obtained from published articles, Supplementary Materials, and publicly available trial registry reports cited in the manuscript. The extracted analytic dataset, source-level extraction ledger, dose-documentation file, contemporaneous PROSPERO version 1.0 record, and executable Python code used to reproduce the pooled estimates and sensitivity analyses are openly available on Zenodo at DOI: 10.5281/zenodo.21481214 (https://zenodo.org/records/21481214; accessed on 4 August 2026). All other relevant data are included in the article and its Supplementary Materials. Further inquiries can be directed to the corresponding author.

## Supplementary Materials

The following supporting information can be downloaded at https://www.mdpi.com/article/10.3390/jcm15166144/s1: Table S1, Initial and updated search strategies and records retrieved by information source; Table S2, Risk-of-bias methodology and domain-level judgments; Table S3, Detailed GRADE evidence profile; Table S4, Detailed trial characteristics, analytic grouping, and participant-outcome mapping; Table S5, PRISMA 2020 checklist; Table S6, Ocular-safety proportion analyses Panel A: aggregate central-injection proportions, exact primary analysis. Panel B: retinal-detachment estimates under alternative methods; Table S7, Dependence-aware primary and sensitivity analyses for pooled functional outcomes; Table S8, Heterogeneity-estimator and robustness sensitivity analyses for pooled functional outcomes; Table S9, Trial-level ocular adverse-event counts underlying the aggregate safety proportions; Table S10, Administered-dose documentation and per-eye calculations; Table S11, Comparator-stratified cohort estimates and external-control exclusion; Table S12, Microperimetry endpoint-compatibility assessment for quantitative synthesis; Table S13, Protocol amendments and post-registration implementation decisions; Figure S1, Low-luminance visual acuity responder analysis.

## Abbreviations

The following abbreviations are used in this manuscript:

AAV	adeno-associated virus
BCVA	best-corrected visual acuity
CENTRAL	Cochrane Central Register of Controlled Trials
CI	confidence interval
DL	DerSimonian–Laird
ETDRS	Early Treatment Diabetic Retinopathy Study
FDA	Food and Drug Administration
GLMM	generalized linear mixed model
GRADE	Grading of Recommendations Assessment, Development and Evaluation
HKSJ	Hartung–Knapp–Sidik–Jonkman
IOP	intraocular pressure
LLVA	low-luminance visual acuity
MAIA	Macular Integrity Assessment
MD	mean difference
MRS	mean retinal sensitivity
MTD	maximum tolerated dose
OHT	ocular hypertension
PRISMA	Preferred Reporting Items for Systematic Reviews and Meta-Analyses
PROSPERO	International Prospective Register of Systematic Reviews
REML	restricted maximum likelihood
RoB 2	Risk of Bias 2
ROBINS-I	Risk Of Bias In Non-randomized Studies of Interventions
RPGR	retinitis pigmentosa GTPase regulator
RPE	retinal pigment epithelium
RR	risk ratio
SAE	serious adverse event
VMA	vision-guided mobility assessment
WHO ICTRP	World Health Organization International Clinical Trials Registry Platform
XLRP	X-linked retinitis pigmentosa.
