# Supplementary material for: Functional Outcomes and Ocular Safety of Subretinal AAV-RPGR Gene Therapy for RPGR-Associated X-Linked Retinitis Pigmentosa: A Systematic Review and Meta-Analysis
Source: J Clin Med. 2026 Aug 7;15(16):6144. doi: 10.3390/jcm15166144 (PMC13513658; doi:10.3390/jcm15166144)
Supplement: Supplementary file 1 [file jcm-15-06144-s001.zip › jcm-4425423-supplementary.pdf]

Supplementary Material

Functional Outcomes and Ocular Safety of Subretinal AAV-RPGR Gene Therapy for RPGR-Associated X-Linked Retinitis Pigmentosa: A Systematic Review and Meta-Analysis

Carlos Roberto Montes-de Oca-Saucedo[1],*, José Antonio Garza-Cruz[1], Bruno Briceño-Villardaga[1], Ingrid Vanessa Infante-Lee[1], Rafael Chavarría-Rojas[1], Natalie Carled Bermejo-Valero[1], Adolfo Soto-Domínguez[1], Neeran Narainswami[2]

[1] Department of Histology, Faculty of Medicine, Universidad Autónoma de Nuevo León, Monterrey 64460, Nuevo León, Mexico; carlos.montess@uanl.edu.mx (C.R.M.-d.-O.-S.); antonio.garzacr@uanl.edu.mx (J.A.G.-C.); bruno.bricenov@uanl.edu.mx (B.B.-V.); ingrid.infantel@uanl.edu.mx (I.V.I.-L.); rc8453529@gmail.com (R.C.-R.); carled.bermejo@gmail.com (N.C.B.-V.); adolfo.sotodmn@uanl.edu.mx (A.S.-D.)

[2] Division of Ophthalmology, Red Cross War Memorial Children's Hospital and Groote Schuur Hospital, University of Cape Town, Cape Town, South Africa; neeran.narainswami@uct.ac.za (N.N.)

*Correspondence: carlos.montess@uanl.edu.mx; Tel.: +52 8116114237

Supplementary contents

Table S1. Initial and updated search strategies and records retrieved by information source
Table S2. Risk-of-bias methodology and domain-level judgments
Table S3. Detailed GRADE evidence profile
Table S4. Detailed trial characteristics, analytic grouping, and participant–outcome mapping
Table S5. PRISMA 2020 checklist
Table S6. Ocular-safety proportion analyses
    Panel A: aggregate central-injection proportions, exact primary analysis
    Panel B: retinal-detachment estimates under alternative methods
Table S7. Dependence-aware primary and sensitivity analyses for pooled functional outcomes
Table S8. Heterogeneity-estimator and robustness sensitivity analyses for pooled functional outcomes
Table S9. Trial-level ocular adverse-event counts underlying the aggregate safety proportions
Table S10. Administered-dose documentation and per-eye calculations
Table S11. Comparator-stratified cohort estimates and external-control exclusion
Table S12. Microperimetry endpoint-compatibility assessment for quantitative synthesis
Table S13. Protocol amendments and post-registration implementation decisions
Figure S1. Low-luminance visual acuity responder analysis

Table S1. Initial and updated search strategies and records retrieved by information source

A. Initial formal search

The initial formal search was completed and archived in Rayyan on 8 October 2025. The following free-text strategy was applied across the searched sources, using the Boolean syntax supported by each interface:

(("retinitis pigmentosa" OR "x-linked retinitis pigmentosa" OR "XLRP" OR "retinitis" OR "x-linked")) AND ("RPGR" OR "GTPase regulator" OR "retinitis pigmentosa GTPase regulator") AND (("gene therapy" OR "gene augmentation" OR "gene replacement" OR "gene transfer" OR "adeno-associated virus" OR "AAV" OR "genetic therapy"))

Information source	Exact date	Records retrieved	Handling
PubMed / MEDLINE	8 October 2025	113	Imported into Rayyan
Web of Science Core Collection	8 October 2025	370	Imported into Rayyan
Embase	8 October 2025	260	Imported into Rayyan
Scopus	8 October 2025	154	Imported into Rayyan
ClinicalTrials.gov	8 October 2025	16	Imported into Rayyan
CENTRAL	8 October 2025	12	Reviewed outside Rayyan; no additional eligible study
WHO ICTRP	8 October 2025	42 records / 21 trials	Reviewed outside Rayyan; no additional eligible study

B. Platform-specific updated search

Each source was rerun from inception on 16–17 July 2026 using the platform-specific strategy shown below. No language or publication-date restrictions were applied.

Information source (platform)	Search fields / vocabulary	Exact updated-search strategy	Updated search date; records
PubMed / MEDLINE (NLM)	MeSH and Title/Abstract [tiab]	("Retinitis Pigmentosa"[Mesh] OR "retinitis pigmentosa"[tiab] OR "X-linked retinitis pigmentosa"[tiab] OR "X linked retinitis pigmentosa"[tiab] OR XLRP[tiab] OR retinitis[tiab] OR "x-linked"[tiab]) AND (RPGR[tiab] OR "GTPase regulator"[tiab] OR "retinitis pigmentosa GTPase regulator"[tiab]) AND ("Genetic Therapy"[Mesh] OR "Dependovirus"[Mesh] OR "gene therapy"[tiab] OR "gene therapies"[tiab] OR "genetic therapy"[tiab] OR "genetic therapies"[tiab] OR "gene augmentation"[tiab] OR "gene replacement"[tiab] OR "gene transfer"[tiab] OR "adeno-associated virus"[tiab] OR "adeno associated virus"[tiab] OR AAV[tiab] OR "cotoretigene toliparvovec"[tiab] OR "botaretigene sparoparvovec"[tiab] OR "AGTC-501"[tiab] OR "laruparetigene zovaparvovec"[tiab] OR "AAV8-coRPGR"[tiab] OR "AAV5-hRKp.RPGR"[tiab] OR "rAAV2tYF-GRK1-RPGR"[tiab] OR rAAV2tYF[tiab] OR BIIB112[tiab] OR "BIIB-112"[tiab])	16 July 2026 n = 123

Information source (platform)	Search fields / vocabulary	Exact updated-search strategy	Updated search date; records
Embase.com (Elsevier)	Emtree (/exp) and Title/Abstract/Key word :ti,ab,kw	('retinitis pigmentosa'/exp OR 'retinitis pigmentosa':ti,ab,kw OR 'x-linked retinitis pigmentosa':ti,ab,kw OR 'x linked retinitis pigmentosa':ti,ab,kw OR xlrp:ti,ab,kw OR retinitis:ti,ab,kw OR 'x-linked':ti,ab,kw) AND (rpgr:ti,ab,kw OR 'gtpase regulator':ti,ab,kw OR 'retinitis pigmentosa gtpase regulator':ti,ab,kw) AND ('gene therapy'/exp OR 'adeno associated virus'/exp OR 'gene therapy':ti,ab,kw OR 'gene therap*':ti,ab,kw OR 'genetic therapy':ti,ab,kw OR 'genetic therap*':ti,ab,kw OR 'gene augmentation':ti,ab,kw OR 'gene replacement':ti,ab,kw OR 'gene transfer':ti,ab,kw OR 'adeno-associated virus':ti,ab,kw OR 'adeno-associated virus*':ti,ab,kw OR 'adeno associated virus':ti,ab,kw OR 'adeno associated virus*':ti,ab,kw OR aav:ti,ab,kw OR 'cotoretigene toliparvovec':ti,ab,kw OR 'botaretigene sparoparvovec':ti,ab,kw OR 'agtc-501':ti,ab,kw OR 'laruparetigene zovaparvovec':ti,ab,kw OR 'aav8-corpgr':ti,ab,kw OR 'aav5-hrkp.rpgr':ti,ab,kw OR 'raav2tyf-grk1-rpgr':ti,ab,kw OR raav2tyf:ti,ab,kw OR biib112:ti,ab,kw OR 'biib-112':ti,ab,kw)	17 July 2026 n = 263
Web of Science Core Collection (Clarivate)	Topic field TS= (title, abstract, author keywords, Keywords Plus)	TS=("retinitis pigmentosa" OR "X-linked retinitis pigmentosa" OR XLRP) AND TS=(RPGR OR "retinitis pigmentosa GTPase regulator") AND TS=("gene therap*" OR "gene augmentation" OR "gene replacement" OR "gene transfer" OR "adeno-associated virus*" OR "adeno associated virus*" OR AAV OR "cotoretigene toliparvovec" OR "botaretigene sparoparvovec" OR "AGTC-501" OR rAAV2tYF)	17 July 2026 n = 265
Scopus (Elsevier)	TITLE-ABS-KEY	TITLE-ABS-KEY("retinitis pigmentosa" OR "X-linked retinitis pigmentosa" OR "X linked retinitis pigmentosa" OR XLRP OR retinitis OR "x-linked") AND TITLE-ABS-KEY(RPGR OR "GTPase regulator" OR "retinitis pigmentosa GTPase regulator") AND TITLE-ABS-KEY("gene therapy" OR "gene therap*" OR "genetic therapy" OR "genetic therap*" OR "gene augmentation" OR "gene replacement" OR "gene transfer" OR "adeno-associated virus" OR "adeno-associated virus*" OR "adeno associated virus" OR "adeno associated virus*" OR AAV OR "cotoretigene toliparvovec" OR "botaretigene sparoparvovec" OR "AGTC-501" OR "laruparetigene zovaparvovec" OR "AAV8-coRPGR" OR "AAV5-hRKp.RPGR" OR "rAAV2tYF-GRK1-RPGR" OR rAAV2tYF OR BIIB112 OR "BIIB-112")	17 July 2026 n = 172

**Information source (platform)**	**Search fields / vocabulary**	**Exact updated-search strategy**	**Updated search date; records**
CENTRAL (Cochrane Library)	MeSH and Title/Abstract/Keyword :ti,ab,kw	([mh "Retinitis Pigmentosa"] OR "retinitis pigmentosa":ti,ab,kw OR "X-linked retinitis pigmentosa":ti,ab,kw OR XLRP:ti,ab,kw) AND (RPGR:ti,ab,kw OR "retinitis pigmentosa GTPase regulator":ti,ab,kw) AND ([mh "Genetic Therapy"] OR [mh Dependovirus] OR "gene therap*":ti,ab,kw OR "gene augmentation":ti,ab,kw OR "gene replacement":ti,ab,kw OR "gene transfer":ti,ab,kw OR "adeno-associated virus*":ti,ab,kw OR "adeno associated virus*":ti,ab,kw OR AAV:ti,ab,kw OR "cotoretigene toliparvovec":ti,ab,kw OR "botaretigene sparoparvovec":ti,ab,kw OR "AGTC-501":ti,ab,kw OR rAAV2tYF:ti,ab,kw)	17 July 2026<br>n = 13
ClinicalTrials.gov	Expert Search: ConditionSearch, OtherTerms, InterventionSearch	AREA[ConditionSearch]("retinitis pigmentosa") AND (AREA[OtherTerms](RPGR OR XLRP OR "retinitis pigmentosa GTPase regulator") OR AREA[InterventionSearch](RPGR OR "gene therapy" OR AAV OR "cotoretigene toliparvovec" OR "botaretigene sparoparvovec" OR "AGTC-501" OR rAAV2tYF))	17 July 2026<br>n = 18
WHO ICTRP Search Portal	Standard Search	("retinitis pigmentosa" OR "X-linked retinitis pigmentosa" OR XLRP) AND (RPGR OR "retinitis pigmentosa GTPase regulator") AND ("gene therapy" OR AAV OR "cotoretigene toliparvovec" OR "botaretigene sparoparvovec" OR "AGTC-501" OR rAAV2tYF)	17 July 2026<br>47 records / 26 trials

Notes. The initial search date was reconstructed from the dated Rayyan record and contemporaneous project documentation. PubMed/MEDLINE, Web of Science, Embase, Scopus, and ClinicalTrials.gov formed the Rayyan deduplication workflow; CENTRAL and WHO ICTRP were reviewed separately. The updated search identified the LUMEOS registry-results report, which was included in the narrative synthesis only; no additional study entered the quantitative synthesis. Reference-list and forward citation searching were also performed.

Table S2 — Risk-of-bias methodology and domain-level judgments

S2.1 Tool selection by contributed result

Risk-of-bias tools were selected according to the design of each contributed result; assessments could therefore differ by outcome within the same study.

Contributed result	Design of that result	Tool
Lam 2024 (XIRIUS Part 2) — microperimetry, BCVA, LLVA	Randomized 1:1:1 with concurrent untreated control	RoB 2
XIRIUS Part 1 (Cehajic-Kapetanovic 2020 / von Krusenstiern 2023), one study	Open-label dose-escalation; untreated fellow-eye control (external XOLARIS subgroup excluded from all quantitative synthesis)	ROBINS-I
Yang 2025 (HORIZON) — microperimetry, BCVA	Open-label dose-escalation; fellow-eye control	ROBINS-I
Michaelides 2024 — BCVA efficacy	Pooled non-randomized escalation + randomized expansion vs pre-treatment deferred control	ROBINS-I
Non-comparative safety proportions from the four programs contributing quantitative safety data	Non-comparative	Review-specific five-domain framework for non-comparative safety outcomes

Michaelides was assessed with ROBINS-I because the pooled efficacy estimate combined non-randomized escalation with randomized expansion participants.

S2.2 Per-outcome application

Assessments were outcome-specific, and each study was evaluated only for the outcomes it contributed. Non-comparative safety outcomes were assessed separately using the framework described in Section S2.5.

S2.3 Traffic-light matrix

RoB 2 (randomized comparison)

Study	D1 Randomization	D2 Deviations	D3 Missing data	D4 Measurement	D5 Reported result	Overall
Lam 2024	Low	Low	Some concerns	Some concerns	Low	Some concerns

ROBINS-I (non-randomized comparisons)

Study	D1 Conf.	D2 Select.	D3 Class.	D4 Devia.	D5 Missing	D6 Measure	D7 Report	Overall
XIRIUS Part 1 (Cehajic 2020 / von Krusenstiern 2023)	Serious	Moderate	Low	Moderate	Moderate	Serious	Moderate	Serious
Yang 2025 (HORIZON)	Serious	Moderate	Low	Moderate	Moderate	Moderate	Moderate	Serious
Michaelides 2024 (efficacy, pooled)	Moderate	Moderate	Low	Moderate	Moderate	Moderate	Low	Moderate

S2.4 Domain-level rationale

Lam 2024 — RoB 2. D1 Low: 1:1:1 random allocation with concealed sequence; study eye also randomized and fixed after allocation (NCT03116113). D2 Low: intention-to-treat analysis, no eye re-designation, no crossover. D3 Some concerns: three of 29 participants withdrew because of COVID-19-related travel restrictions (high-dose n=2, low-dose n=1). Although the reasons were unrelated to outcome status, the small cohort increases the potential impact of the missing observations. D4 Some concerns: outcome assessors masked to study eye, dose, and treatment, yet participants were unmasked to treatment status, allowing performance bias on effort-dependent functional endpoints. D5 Low: pre-registered primary endpoint and pre-specified analysis (Boschloo test with Berger-Boos correction).

XIRIUS Part 1 (Cehajic-Kapetanovic 2020 / von Krusenstiern 2023) — ROBINS-I, one study. D1 Serious: open-label dose-escalation with no concurrent randomized control; comparisons rest on untreated fellow eyes only. The external XOLARIS natural-history subgroup reported in the source was excluded from all quantitative synthesis in this review. D2 Moderate: sequential cohort enrolment by clinical judgment. D3 Low: per-patient vector dose documented. D4 Moderate: dose-related intraocular inflammation managed with steroids transiently depressed microperimetry in higher-dose cohorts. D5 Moderate: small n; some microperimetry acquisitions were unavailable or unreliable, and their handling was incompletely described in the source (rather than uniformly excluded). D6 Serious: open-label, effort-dependent microperimetry, with some acquisitions unavailable or unreliable. D7 Moderate: results split across two publications and timepoints.

Yang 2025 (HORIZON) — ROBINS-I. D1 Serious: open-label dose-escalation; study-eye versus untreated fellow-eye comparison; central and peripheral injection routes differ structurally. D2 Moderate: investigator-determined dosing and enrolment. D3 Low: vector dose documented. D4 Moderate: steroid management and dose-related retinal pigment epithelium changes at the highest dose. D5 Moderate: missing values excluded rather than imputed; small n. D6 Moderate: open-label, effort-dependent microperimetry, partly offset by the stricter two-consecutive-visit responder criterion. D7 Moderate: multiple dosing groups and timepoints.

Michaelides 2024 (efficacy, pooled) — ROBINS-I. D1 Moderate: a randomized, deferred-treatment concurrent control mitigates confounding, but the reported efficacy estimate pools randomized expansion with non-randomized escalation participants and uses control data only from the pre-treatment window, reintroducing temporal and selection confounding. D2 Moderate: mixed randomized and non-randomized participants. D3 Low: doses documented; one participant inadvertently received the high dose (analysed by assigned dose for efficacy, actual dose for safety). D4 Moderate: the dosing deviation above; steroid prophylaxis. D5 Moderate: four control participants discontinued before treatment. D6 Moderate: open-label, effort-dependent functional testing. D7 Low: pre-registered, with safety primary and efficacy a pre-specified secondary.

S2.5 Risk of bias for non-comparative safety outcomes (review-specific framework).

Because RoB 2 and ROBINS-I address comparative intervention effects, non-comparative safety proportions were assessed with a review-specific, nonvalidated five-domain framework specified before reassessment: selection, ascertainment, definition/classification, completeness, and selective reporting. Domains were rated low risk, some concerns, or high risk. Overall risk was low when all domains were low, high when any domain was high or multiple concerns were likely to distort the estimate, and some concerns otherwise. Small samples and sparse events were considered under GRADE imprecision.

Domain criteria were: selection, inclusion of all treated eyes without outcome-related post-intervention exclusions; ascertainment, prospective scheduled surveillance; definition/classification, explicit and reproducible event criteria; completeness, a clear denominator and adequate follow-up; and selective reporting, concordance between the registry or protocol and the published report.

Safety outcome	(1) Selection	(2) Ascertainment	(3) Definition / classification	(4) Completeness	(5) Selective reporting [a]	Overall
Intraocular inflammation	Low	Low	Some concerns [b]	Some concerns [c]	Low [a]	**Some concerns**
Any ocular serious adverse event	Low	Low	Low	Some concerns [c]	Low [a]	**Some concerns**
Retinal detachment	Low	Low	Low	Some concerns [c]	Low [a]	**Some concerns**

For intraocular inflammation, HORIZON was not assessed because it did not contribute a deduplicated participant-level composite to the synthesized outcome; its explicitly reported component terms were retained separately (Table S9) and were not combined to derive that composite.

Selective-reporting crosswalk (registry / protocol / publication).

Cohort	Registry	Safety as registered objective	Contributing treated population in this review	Observation window reported
XIRIUS Part 1	NCT03116113	TEAE/DLT (primary)	All 18 treated	24 months (all completed)
XIRIUS Part 2	NCT03116113	TEAE incidence (primary)	All 23 treated	12 months (3 COVID discontinuations)
Botaretigene	NCT03252847	Safety (primary)	All 32 immediately treated	52 wk (2 discontinuations)
HORIZON	NCT03316560	TEAE/SAE (primary/co-primary)	29 treated (21 central)	24 months

Safety was prospectively ascertained in open-label studies; central blinded adjudication of individual adverse events was not reported.

[a] Selective reporting was rated low because safety was a registered primary or co-primary objective and the full contributing treated population was reported. HORIZON's lack of a deduplicated inflammation composite reflects the construct used in this review rather than omission of a prespecified trial outcome.

[b] Inflammation classification was open-label and investigator-determined; definitions and preferred terms differed between programs.

[c] Completeness raised some concerns because three XIRIUS Part 2 participants and two botaretigene participants discontinued before the intended safety window.

Table S3. Detailed GRADE Evidence Profile

Certainty was rated per outcome using GRADE and design-specific risk-of-bias assessments, following the ROBINS-I-integrated approach. Functional outcomes are reported as mean differences and safety outcomes as exact treated-eye proportions; random-effects models were retained as sensitivity analyses.

(A) Mesopic retinal sensitivity — 6 months

Domain	Judgment	Rationale
Risk of bias	Downgrade 1	The estimate is carried mainly by open-label, non-randomized dose-escalation cohorts (ROBINS-I serious), with one small randomized comparison (Lam 2024, RoB 2 some concerns). Confounding inherent to escalation designs and unmasked, effort-dependent microperimetry place the body at serious overall risk.
Inconsistency	No downgrade	Under independent analytic cohort-level aggregation, heterogeneity was low-to-moderate (I^2 = 28%; τ^2 = 0.18 dB^2) with effects in the same direction across cohorts; the spread is consistent with sampling variation rather than conflicting findings.
Indirectness	No downgrade	Mesopic MAIA microperimetry is the pre-specified, mechanistically aligned functional endpoint, measured in the target population with comparable acquisitions.
Imprecision	Downgrade 2	Under the primary independent analytic cohort-level model only three independent cohorts contribute; the 95% interval (−0.88 to +3.09 dB) spans possible worsening, no effect, and benefit of uncertain clinical importance. This is very serious imprecision.
Publication bias	Undetected	With fewer than ten studies a funnel-based test is uninformative; reporting bias is plausible but not applied as a formal downgrade.
**Final certainty**	**Very low (⊕◯◯◯)**	**Very low: risk of bias and very serious imprecision**

(B) Mesopic retinal sensitivity — 12 months

Domain	Judgment	Rationale
Risk of bias	Downgrade 1	One randomized concurrent-control cohort (XIRIUS Part 2) and one open-label fellow-eye cohort (HORIZON) contributed; the limitations arising from the nonrandomized component therefore remained.
Inconsistency	No downgrade	Under cohort-level aggregation heterogeneity was zero (I^2 = 0%); however, only two independent analytic cohorts contribute.
Indirectness	No downgrade	Same pre-specified, mechanistically aligned functional endpoint as at 6 months.
Imprecision	Downgrade 2	Only two independent analytic cohorts contribute (df = 1), and the 95% interval (−3.99 to +5.94 dB) spans markedly different clinical conclusions; the estimate is exploratory and highly unstable. This is very serious imprecision.
Publication bias	Undetected	Too few studies for formal assessment.
**Final certainty**	**Very low (⊕◯◯◯)**	**Very low: risk of bias and very serious imprecision**

(C) Best-corrected visual acuity — 6 months

Domain	Judgment	Rationale
Risk of bias	Downgrade 1	The body combined one randomized cohort (XIRIUS Part 2), one open-label fellow-eye cohort (XIRIUS Part 1), and one mixed-design deferred-control cohort (Botaretigene). BCVA is less effort-dependent than microperimetry, but design confounding persists.
Inconsistency	No downgrade	Under independent analytic cohort-level aggregation, heterogeneity was moderate (I^2 = 64%; τ^2 = 3.62 ETDRS letters²). Cohort estimates ranged from near-null or slightly negative to positive; with only three cohorts, inconsistency was considered insufficiently characterized for a separate downgrade.
Indirectness	No downgrade	BCVA is a direct, patient-important outcome. A ceiling effect in RPGR limits its sensitivity to change but does not bias the treated-versus-control comparison.
Imprecision	Downgrade 2	Only three independent analytic cohorts contributed, and the 95% CI (−4.97 to +6.85 ETDRS letters) spans possible worsening, no effect, and improvement. This was judged very serious imprecision.
Publication bias	Undetected	Fewer than ten studies.
**Final certainty**	**Very low (⊕◯◯◯)**	**Very low: risk of bias and very serious imprecision**

(D) Low-luminance visual acuity gain ≥10 letters — 6 months

Domain	Judgment	Rationale
Risk of bias	Downgrade 1	A two-study estimate from one RCT (some concerns) and one non-randomized cohort (serious); threshold-based responder counting inherits the open-label limitations of its inputs.
Inconsistency	No downgrade	$I^2 = 0\%$, although with only two studies inconsistency is barely estimable.
Indirectness	No downgrade	Low-luminance acuity is a relevant functional outcome in rod-cone degeneration.
Imprecision	Downgrade 2	The pooled risk ratio (3.83) does not reach significance under the primary Mantel–Haenszel model: the 95% interval runs from 0.94 to 15.57, crossing the null, on a very small event count across two studies, and the result is sensitive to a single untreated-eye event. This is very serious imprecision.
Publication bias	Undetected	Two studies only.
**Final certainty**	**Very low (⊕◯◯◯)**	**Very low: risk of bias and very serious imprecision**

(E) Intraocular inflammation — participant-level composite (three derivable cohorts)

Domain	Judgment	Rationale
Risk of bias	Downgrade 1	Single-arm ascertainment across open-label trials, with inflammation grading and reporting not standardized between programs.
Inconsistency	Downgrade 1	Cohort-level inflammation proportions varied materially across programs (XIRIUS Part 1 66.7%, XIRIUS Part 2 95.7%, Botaretigene 78.1%), with differing event definitions and ascertainment frameworks; HORIZON reported overlapping inflammatory preferred terms without a deduplicated central composite and was summarized descriptively.
Indirectness	No downgrade	A directly measured ocular adverse event in the treated population.
Imprecision	No downgrade	The aggregate was restricted to three cohorts reporting an explicit participant-level composite (59/73; exact 95% CI, 69.9% to 89.1%), consistently indicating a high incidence of intraocular inflammation. The broad random-effects logit HKSJ sensitivity interval reflects small-sample uncertainty and method sensitivity rather than imprecision of the primary exact estimate.
Publication bias	Undetected	Fewer than ten cohorts.
**Final certainty**	**Low (⊕⊕◯◯)**	**Low: risk of bias and inconsistency.**

(F) Any ocular serious adverse event — central-injection treated-eye proportion

Domain	Judgment	Rationale
Risk of bias	Downgrade 1	Single-arm, open-label ascertainment without a comparator.
Inconsistency	No downgrade	No major directional inconsistency was evident across the central-injection cohorts; between-program variability was examined in the random-effects logit sensitivity analysis.
Indirectness	No downgrade	Directly observed ocular harm in the treated eyes.
Imprecision	Downgrade 1	A wide exact (Clopper–Pearson) 95% interval (10.1 to 26.2%) on a small denominator.
Publication bias	Undetected	Too few studies for formal testing.
**Final certainty**	**Low (⊕⊕◯◯)**	**Low: risk of bias and imprecision.**

Primary estimand: 16/94 events; 17.0% (95% CI, 10.1 to 26.2). Excluding one ocular serious adverse event attributed by the investigators to natural disease progression yielded 15/94 events (16.0%; 95% CI, 9.2 to 25.0) as a sensitivity analysis.

(G) Retinal detachment, central injection — proportion

Domain	Judgment	Rationale
Risk of bias	Downgrade 1	Single-arm ascertainment, mitigated somewhat because detachment is an objective, hard endpoint less prone to measurement error than functional outcomes.
Inconsistency	No downgrade	Low between-study spread once peripheral-injection cohorts are removed.
Indirectness	No downgrade	Restricting to the central-injection route sharpens rather than weakens applicability, isolating the surgical risk of central subretinal delivery from the peripheral-procedure signal.

**Domain**	**Judgment**	**Rationale**
Imprecision	Downgrade 1	Only four events occurred among 94 centrally injected treated eyes, and the exact (Clopper–Pearson) 95% CI (1.2 to 10.5%) permits materially different estimates of absolute surgical risk.
Publication bias	Undetected	Fewer than ten cohorts.
**Final certainty**	**Low (⊕⊕◯◯)**	**Low: risk of bias and imprecision.**

Table S4. Detailed trial characteristics, analytic grouping, and participant–outcome mapping

XIRIUS Part 1 — cotoretigene toliparvovec (AAV8.coRPGR / BIIB112)

Reports / registry record	Cehajic-Kapetanovic 2020 (6-month report); von Krusenstiern 2023 (retinal-sensitivity report). NCT03116113 (XIRIUS Part 1).
Vector / cassette	AAV8.coRPGR; codon-optimized RPGR-ORF15; rhodopsin kinase (GRK1) promoter.
Design	Phase 1/2, open-label, dose escalation.
Analytic cohort	One analytic cohort reported in two publications.
Enrolled participants	18.
Treated participants / eyes	18 participants / 18 treated eyes (one eye per participant).
Comparator	Untreated fellow eye of the same participant. The external XOLARIS natural-history subgroup reported in the source was not used as a comparator in any quantitative analysis.
Delivery / target	Subretinal; central / posterior pole.
Functional outcomes contributed	Mesopic MAIA microperimetry (Month 6); BCVA (Month 6); LLVA responder (Months 6 and 12). Month-6 microperimetry and BCVA were computed from the published participant-level bilateral data as observed treated-minus-fellow-eye change contrasts.
Safety population contributed	18 treated eyes (central injection).
Follow-up	24 months (functional analyses at Months 6 and 12).
Overlap handling	The two reports describe the same 18 participants and were treated as one study, one analytic cohort, and one safety denominator. von Krusenstiern 2023 provided the later retinal-sensitivity and LLVA data.

XIRIUS Part 2 — cotoretigene toliparvovec (AAV8.coRPGR / BIIB112)

Reports / registry record	Lam 2024. NCT03116113 (XIRIUS phase 2/3 dose-expansion part).
Vector / cassette	AAV8.coRPGR; codon-optimized RPGR-ORF15; rhodopsin kinase (GRK1) promoter.
Design	Phase 2/3, randomized 1:1:1 (low dose, high dose, untreated control).
Analytic cohort	Separate, non-overlapping analytic cohort from XIRIUS Part 1.
Enrolled participants	32 randomized (safety analysis set, n = 32); intention-to-treat analysis set, n = 29. Three participants were randomized under an earlier two-group protocol version and are included in the safety analysis set only.
Treated participants / eyes	23 treated participants / 23 treated eyes in the safety analysis set (low dose 11, high dose 12); 20 treated participants in the intention-to-treat set (10 low, 10 high).
Comparator	Concurrent randomized untreated control (n = 9); outcome-specific denominators are listed below.
Delivery / target	Subretinal; central macula, after pars plana vitrectomy.
Functional outcomes contributed	Mesopic MAIA microperimetry (Months 6 and 12); BCVA (Month 6); LLVA responder (Months 6 and 12).
Safety population contributed	23 treated eyes (central injection).
Follow-up	12 months.
Overlap handling	Within Part 2, the two treated arms were combined and the shared randomized control was counted once.

Botaretigene sparoparvovec — AAV5-hRKp.RPGR (MGT-RPGR-011)

Reports / registry record	Michaelides 2024. NCT03252847.
Vector / cassette	AAV5-hRKp.RPGR; RPGR-ORF15 with a 378-base-pair in-frame deletion; human rhodopsin kinase promoter.
Design	Phase 1/2, open-label dose escalation and dose confirmation with a randomized, controlled dose-expansion phase (immediate versus deferred treatment).
Analytic cohort	One mixed-design efficacy cohort (dose escalation plus randomized dose expansion).
Enrolled participants	49 enrolled: dose escalation, 10 adults; dose confirmation, 3 paediatric participants; dose expansion, 36 randomized 1:1:1 to immediate or deferred treatment.
Treated participants / eyes	45 participants received vector at some point during the study (safety analysis set). The immediately treated cohorts contributing to this review are 32 participants / 32 treated eyes (low dose 11, intermediate dose 17, high dose 4).
Comparator	Deferred-treatment control group, n = 13, during the concurrent Week-26 comparison window.
Delivery / target	Subretinal; central / posterior pole, after pars plana vitrectomy.
Functional outcomes contributed	BCVA (Month 6 / Week 26), pooling immediately treated low- and intermediate-dose participants against the deferred-control window; 25 treated participants and 13 controls had evaluable Week-26 BCVA and formed the analytic denominator.Retinal sensitivity was assessed by static photopic perimetry and scotopic microperimetry and therefore did not enter the mesopic MAIA microperimetry synthesis (Table S12).
Safety population contributed	32 immediately treated eyes with a uniform post-treatment observation window. The 13 deferred-control participants were not included in the pooled safety denominator because treatment began after Week 26 and comparable 52-week post-treatment safety data were unavailable.
Follow-up	Week 26 (control comparison) and Week 52 (treated participants).
Overlap handling	Escalation and expansion participants are distinct individuals pooled once into a single cohort estimate. The 13 deferred participants served as the concurrent control during the Week-26 comparison and were themselves treated after Week 26; they are counted only in the control column for that window and are never added to the treated-eye denominator used here.

HORIZON — AGTC-501 / laruparetigene zovaparvovec (rAAV2tYF-GRK1-RPGR)

Reports / registry record	Yang 2025. NCT03316560.
Vector / cassette	rAAV2tYF-GRK1-RPGR; full-length RPGR; GRK1 (rhodopsin kinase) promoter.
Design	Phase 1/2, open-label, dose escalation.
Analytic cohort	One analytic cohort, with central- and peripheral-injection subgroups reported separately.
Enrolled participants	29 treated participants: 21 received central injections and 8 received peripheral injections.

Treated participants / eyes	29 participants / 29 treated eyes (one eye per participant).
Comparator	Untreated fellow eye of the same participant.
Delivery / target	Subretinal; injection sites initially peripheral and transitioned to the central macula with successive groups.
Functional outcomes contributed	Mesopic MAIA microperimetry at Months 6 and 12 from tolerated-dose central Groups 4 and 5. Group 6, above the program-specific MTD, was descriptive only and excluded from pooled efficacy; Groups 1–2 contributed to safety only.
Safety population contributed	21 central eyes in the primary safety analysis; 8 peripheral eyes were added only to the all-groups sensitivity analysis.
Follow-up	6–24 months.
Overlap handling	Central and peripheral subgroups were mutually exclusive; HORIZON entered once per analysis set.

LUMEOS — botaretigene sparoparvovec (MGT-RPGR-021)

Reports / registry record	Structured registry-results report, NCT04671433 / NL-OMON52296; study completed 24 June 2024, results posted 26 September 2025.
Vector / cassette	AAV5-hRKp.RPGR; same investigational product as the phase 1/2 botaretigene programme.
Design	Phase 3, randomized, controlled; bilateral treatment.
Enrolled participants	97 enrolled; 95 remained in the principal randomized population after two pre-surgery withdrawals.
Treated participants / eyes	62 participants received immediate bilateral treatment and 33 were assigned to deferred treatment. No LUMEOS participant or eye entered a quantitative denominator.
Comparator	Randomized deferred-treatment control group (n = 33).
Delivery / target	Subretinal, bilateral (both eyes treated).
Functional outcomes contributed	Narrative only: binocular vision-guided mobility, static-perimetry mean retinal sensitivity, BCVA, and LLVA.
Safety population contributed	Narrative only: 62 immediately treated participants; safety was reported bilaterally at the participant level.
Follow-up	Week 52 (primary analysis).
Overlap handling	LUMEOS evaluates the same investigational product as the botaretigene phase 1/2 programme, but enrolled different participants. The quantitative-analysis study set was unchanged by its inclusion.

Participant and eye accounting.

The four quantitative programs enrolled or randomized 128 participants and contributed 102 treated eyes. The 94 centrally injected eyes formed the primary safety denominator; 8 peripheral HORIZON eyes were added only in all-groups sensitivity analyses. Untreated fellow eyes and randomized or deferred controls never entered treated-eye denominators. LUMEOS contributed to narrative synthesis only.

Arm-level denominators contributing to each pooled functional outcome.

Outcome and timepoint	Cohort and arm	n treated / control
Mesopic microperimetry, Month 6	XIRIUS Part 1 — lower dose; higher-tolerated dose	8 / 8; 7 / 7
	XIRIUS Part 2 — lower dose; higher-tolerated dose	9 / 4; 8 / 5
	HORIZON — Group 4; Group 5	7 / 7; 7 / 7
	HORIZON — Group 6 (above MTD, descriptive only)	4 / 4
Mesopic microperimetry, Month 12	XIRIUS Part 2 — lower dose; higher-tolerated dose	8 / 4; 8 / 5
	HORIZON — Group 4; Group 5	7 / 7; 7 / 7
	HORIZON — Group 6 (above MTD, descriptive only)	4 / 4
BCVA, Month 6	XIRIUS Part 1 — lower dose; higher-tolerated dose	10 / 10; 8 / 8
	XIRIUS Part 2 — lower dose; higher-tolerated dose	9 / 5; 8 / 4
	Botaretigene — mixed dose vs deferred control	25 / 13
LLVA responder, Months 6 and 12	XIRIUS Part 1	11 / 11
	XIRIUS Part 2	17 / 9

For fellow-eye comparisons, the control denotes the untreated fellow eye. Shared controls are displayed by arm for descriptive purposes but entered cohort-level analyses once. XIRIUS Part 1 microperimetry denominators were lower because of unavailable or invalid acquisitions (Table S7). Safety denominators and administered doses are reported in Tables S9 and S10, respectively.

Abbreviations: AAV, adeno-associated virus; BCVA, best-corrected visual acuity; gp, genome particles; LLVA, low-luminance visual acuity; MAIA, Macular Integrity Assessment microperimetry; MTD, maximum tolerated dose; vg, vector genomes.

Table S5. PRISMA 2020 Checklist

Section and Topic	Item #	Checklist item	Location where item is reported
**TITLE**			
Title	1	Identify the report as a systematic review.	Title
**ABSTRACT**			
Abstract	2	See the PRISMA 2020 for Abstracts checklist.	Abstract
**INTRODUCTION**			
Rationale	3	Describe the rationale for the review in the context of existing knowledge.	Section 1
Objectives	4	Provide an explicit statement of the objective(s) or question(s) the review addresses.	Section 1
**METHODS**			
Eligibility criteria	5	Specify the inclusion and exclusion criteria for the review and how studies were grouped for the syntheses.	Section 2.2
Information sources	6	Specify all databases, registers, websites, organisations, reference lists and other sources searched or consulted to identify studies. Specify the date when each source was last searched or consulted.	Section 2.3; Table S1
Search strategy	7	Present the full search strategies for all databases, registers and websites, including any filters and limits used.	Section 2.3; Table S1
Selection process	8	Specify the methods used to decide whether a study met the inclusion criteria of the review, including how many reviewers screened each record and each report retrieved, whether they worked independently, and if applicable, details of automation tools used in the process.	Section 2.3
Data collection process	9	Specify the methods used to collect data from reports, including how many reviewers collected data from each report, whether they worked independently, any processes for obtaining or confirming data from study investigators, and if applicable, details of automation tools used in the process.	Section 2.4
Data items	10a	List and define all outcomes for which data were sought. Specify whether all results that were compatible with each outcome domain in each study were sought (e.g. for all measures, time points, analyses), and if not, the methods used to decide which results to collect.	Section 2.5; Table S12
Data items	10b	List and define all other variables for which data were sought (e.g. participant and intervention characteristics, funding sources). Describe any assumptions made about any missing or unclear information.	Section 2.4; Table 1; Tables S4 and S10
Study risk of bias assessment	11	Specify the methods used to assess risk of bias in the included studies, including details of the tool(s) used, how many reviewers assessed each study and whether they worked independently, and if applicable, details of automation tools used in the process.	Section 2.7; Section 3.7; Figure 5; Table S2
Effect measures	12	Specify for each outcome the effect measure(s) (e.g. risk ratio, mean difference) used in the synthesis or presentation of results.	Section 2.6
Synthesis methods	13a	Describe the processes used to decide which studies were eligible for each synthesis (e.g. tabulating the study intervention characteristics and comparing against the planned groups for each synthesis (item #5)).	Sections 2.2, 2.5, and 2.6; Tables S4 and S12
Synthesis methods	13b	Describe any methods required to prepare the data for presentation or synthesis, such as handling of missing summary statistics, or data conversions.	Sections 2.4 and 2.6; Tables S7 and S10
Synthesis methods	13c	Describe any methods used to tabulate or visually display results of individual studies and syntheses.	Section 2.6; Figures 2–4; Figure S1
Synthesis methods	13d	Describe any methods used to synthesize results and provide a rationale for the choice(s). If meta-analysis was performed, describe the model(s), method(s) to identify the presence and extent of statistical heterogeneity, and software package(s) used.	Section 2.6; Tables S6–S8
Synthesis methods	13e	Describe any methods used to explore possible causes of heterogeneity among study results (e.g. subgroup analysis, meta-regression).	Section 2.6; Table S11
Synthesis methods	13f	Describe any sensitivity analyses conducted to assess robustness of the synthesized results.	Section 2.6; Tables S6–S8
Reporting bias assessment	14	Describe any methods used to assess risk of bias due to missing results in a synthesis (arising from reporting biases).	Section 2.6
Certainty assessment	15	Describe any methods used to assess certainty (or confidence) in the body of evidence for an outcome.	Section 2.7; Table S3
**RESULTS**			
Study selection	16a	Describe the results of the search and selection process, from the number of records identified in the search to the number of studies included in the review, ideally using a flow diagram.	Section 3.1; Figure 1
Study selection	16b	Cite studies that might appear to meet the inclusion criteria, but which were excluded, and explain why they were excluded.	Section 3.1; Figure 1
Study characteristics	17	Cite each included study and present its characteristics.	Section 3.1; Table 1; Table S4
Risk of bias in studies	18	Present assessments of risk of bias for each included study.	Section 3.7; Figure 5; Table S2
Results of individual studies	19	For all outcomes, present, for each study: (a) summary statistics for each group (where appropriate) and (b) an effect estimate and its precision (e.g. confidence/credible interval), ideally using structured tables or plots.	Sections 3.2–3.6; Figures 2–4; Figure S1; Tables S4 and S9
Results of syntheses	20a	For each synthesis, briefly summarize the characteristics and risk of bias among contributing studies.	Sections 3.2–3.8; Table 1; Figure 5; Tables S2–S4
Results of syntheses	20b	Present results of all statistical syntheses conducted. If meta-analysis was done, present for each the summary estimate and its precision (e.g. confidence/credible interval) and measures of statistical heterogeneity. If comparing groups, describe the direction of the effect.	Sections 3.2–3.6; Figures 2–4; Figure S1; Table 2; Tables S6–S8

Section and Topic	Item #	Checklist item	Location where item is reported
Results of syntheses	20c	Present results of all investigations of possible causes of heterogeneity among study results.	Sections 3.2, 3.4, and 3.6; Tables S6–S8 and S11
Results of syntheses	20d	Present results of all sensitivity analyses conducted to assess the robustness of the synthesized results.	Sections 3.2, 3.4–3.6; Tables S6–S8;
Reporting biases	21	Present assessments of risk of bias due to missing results (arising from reporting biases) for each synthesis assessed.	Section 3.8; Table S3
Certainty of evidence	22	Present assessments of certainty (or confidence) in the body of evidence for each outcome assessed.	Section 3.8; Table 2; Table S3
**DISCUSSION**			
Discussion	23a	Provide a general interpretation of the results in the context of other evidence.	Section 4
Discussion	23b	Discuss any limitations of the evidence included in the review.	Section 4
Discussion	23c	Discuss any limitations of the review processes used.	Section 4
Discussion	23d	Discuss implications of the results for practice, policy, and future research.	Sections 4 and 5
**OTHER INFORMATION**			
Registration and protocol	24a	Provide registration information for the review, including register name and registration number, or state that the review was not registered.	Section 2.1
Registration and protocol	24b	Indicate where the review protocol can be accessed, or state that a protocol was not prepared.	Section 2.1; Data Availability Statement; Zenodo deposit
Registration and protocol	24c	Describe and explain any amendments to information provided at registration or in the protocol.	Sections 2.1 and 2.8; Table S13
Support	25	Describe sources of financial or non-financial support for the review, and the role of the funders or sponsors in the review.	Funding
Competing interests	26	Declare any competing interests of review authors.	Conflicts of Interest
Availability of data, code and other materials	27	Report which of the following are publicly available and where they can be found: template data collection forms; data extracted from included studies; data used for all analyses; analytic code; any other materials used in the review.	Data Availability Statement; Zenodo DOI: 10.5281/zenodo.21481214

Table S6. Ocular-safety proportion analyses
Panel A. Aggregate central-injection proportions and exact primary estimates

Safety outcome	Events / eyes	Exact binomial, % (95% CI)	Random-effects logit with HKSJ interval, % (95% CI) a
Any ocular serious adverse event d	16 / 94	17.0 (10.1–26.2)	19.6 (7.4–42.7)
Procedure-related retinal complication	8 / 94	8.5 (3.7–16.1)	11.6 (4.1–28.5)
Retinal detachment	4 / 94	4.3 (1.2–10.5)	6.2 (4.3–9.0)
Intraocular inflammation (three derivable cohorts) b	59 / 73	80.8 (69.9–89.1)	79.7 (23.3–98.1)
Anterior chamber cell (reported component term) c	24 / 71	33.8 (23.0–46.0)	—
Elevated IOP / ocular hypertension (reported component term) c	22 / 50	44.0 (30.0–58.7)	—

Notes. The central-injection set comprised XIRIUS Part 1 (18 eyes), XIRIUS Part 2 (23), botaretigene (32), and HORIZON central (21). Exact Clopper–Pearson intervals are the primary estimates.

[a] Random-effects logit estimates in Panel A use HKSJ intervals and are sensitivity analyses for the principal safety outcomes; no model-based estimate was calculated for descriptive component terms.

[b] Inflammation was aggregated only for cohorts reporting an explicit deduplicated composite: XIRIUS Part 1, 12/18; XIRIUS Part 2, 22/23; and botaretigene, 25/32. HORIZON component terms were not summed (Table S9).

[c] Component outcomes include only cohorts reporting explicit counts: anterior chamber cell, 24/71; IOP/OHT, 22/50. Nonreporting cohorts were excluded rather than treated as having zero events.

[d] Excluding one XIRIUS Part 1 ocular SAE attributed to natural disease progression yielded 15/94 (16.0%; 95% CI, 9.2–25.0). All-groups estimates were 19/102 for ocular SAE and 7/102 for retinal detachment.

Table S6, Panel B. Retinal-detachment estimates under alternative estimation methods

Estimation method	Analysis set	Events / eyes	Estimated proportion (%)	95% CI (%)
Exact binomial (Clopper–Pearson) a	Central-injection	4 / 94	4.3	1.2–10.5
Beta-binomial (ML) b	Central-injection	4 / 94	4.3; ρ ≈ 0	Not separately estimated
Random-effects logit (two-stage, Wald-z) c	Central-injection	4 / 94	6.2	2.8–13.2
Binomial-normal GLMM (one-stage) d	Central-injection	4 / 94	4.3	1.6–10.8
Exact binomial (Clopper–Pearson) a	All groups	7 / 102	6.9	2.8–13.6
Beta-binomial (ML) b	All groups	7 / 102	6.9; ρ ≈ 0	Not separately estimated
Random-effects logit (two-stage, Wald-z) c	All groups	7 / 102	9.4	5.0–17.2
Binomial-normal GLMM (one-stage) d	All groups	7 / 102	6.9	3.3–13.7

Central-injection cohorts: XIRIUS Part 1 (1/18), XIRIUS Part 2 (1/23), botaretigene sparoparvovec (1/32), and HORIZON centrally injected eyes (1/21). The all-groups analysis additionally includes the HORIZON peripheral-injection cohort (3/8).

[a] Exact Clopper–Pearson interval; primary method.

[b] Beta-binomial maximum-likelihood model. The dispersion estimate was at the boundary ($\rho \approx 0$); with four cohorts, overdispersion was not identifiable and no separate confidence interval was estimated.

[c] Two-stage DL random-effects logit model with a 0.5 continuity correction and Wald-z interval. HORIZON entered once in each analysis set.

[d] One-stage binomial-normal GLMM. Between-cohort variance was estimated at the boundary ($\tau^2 \approx 0$), and point estimates matched the exact aggregate estimates.

Table S7. Dependence-aware primary and sensitivity analyses for pooled functional outcomes.

Outcome	Analysis	Pooled MD (95% CI) or point-estimate range	P value or range	Note
Mesopic microperimetry, 6 mo	Primary — independent analytic cohort-level, HKSJ (k = 3) [a]	+1.11 (−0.88, +3.09)	0.138	$I^2 = 28\%$; $\tau^2 = 0.18$ dB^2
	Conventional DL normal-approximation (z-based) [e]	+1.11 (+0.25, +1.96)	0.011	Normal-approximation inference; nominal significance is model-dependent
	Descriptive — arm-level (k = 6) [b]	+1.16 (−0.17, +2.48)	0.075	Treats each arm as independent; overstates precision
	Reliability sensitivity (XIRIUS Part 1 excl. C5.3, n = 14) [f]	+1.13 (−0.77, +3.03)	0.124	Excludes the acquisition described as unreliable in the source
	Leave-one-cohort-out [c]	+0.85 to +1.43	0.168–0.395	Not driven by any single cohort
	Paired-eye correlation (ρ = 0 / 0.25 / 0.50 / 0.75) [d]	+1.11 / +1.10 / +1.08 / +1.07	0.138 / 0.132 / 0.123 / 0.112	HORIZON only; XIRIUS Part 1 uses observed paired differences; nonsignificant under HKSJ throughout
Mesopic microperimetry, 12 mo	Primary — independent analytic cohort-level, HKSJ (k = 2) [a]	+0.97 (−3.99, +5.94)	0.243	Exploratory; two cohorts (df = 1); $I^2 = 0\%$
	Conventional DL normal-approximation (z-based) [e]	+0.97 (+0.16, +1.79)	0.019	Normal-approximation inference; nominal significance is model-dependent
	Descriptive — arm-level (k = 4) [b]	+0.97 (−0.10, +2.03)	0.063	Treats each arm as independent; overstates precision
	Paired-eye correlation (ρ = 0 / 0.25 / 0.50 / 0.75) [d]	+0.97 / +0.93 / +0.89 / +0.85	0.243 / 0.232 / 0.212 / 0.188	HORIZON paired only; exploratory (k = 2); nonsignificant under HKSJ throughout
BCVA, 6 mo	Primary — independent analytic cohort-level, HKSJ (k = 3) [a]	+0.94 (−4.97, +6.85)	0.565	$I^2 = 64\%$; $\tau^2 = 3.62$ ETDRS $letters^2$
	Conventional DL normal-approximation (z-based) [e]	+0.94 (−1.75, +3.63)	0.494	Interval narrower than HKSJ; remained nonsignificant
	Descriptive — arm-level (k = 5) [b]	+0.66 (−2.39, +3.71)	0.582	Treats each arm as independent; overstates precision

	Leave-one-cohort-out [c]	−0.41 to +1.88	0.480–0.656	Not driven by any single cohort
	Paired-eye correlation [d]	Not applicable	—	XIRIUS Part 1 contributes an observed participant-level paired difference; no assumed correlation required (HORIZON not in the BCVA pool)

Paired-eye correlation sensitivity — ρ-specific traceability panel.

Outcome	ρ	MD	HKSJ 95% CI	P	τ^2
Micro M6 (HORIZON only; XIRIUS P1 observed)	0.00	+1.11	(−0.88, +3.09)	0.138	0.18
	0.25	+1.10	(−0.81, +3.00)	0.132	0.17
	0.50	+1.08	(−0.72, +2.89)	0.123	0.16
	0.75	+1.07	(−0.61, +2.75)	0.112	0.15
Micro M12 (HORIZON paired; exploratory)	0.00	+0.97	(−3.99, +5.94)	0.243	0.00
	0.25	+0.93	(−3.59, +5.46)	0.232	0.00
	0.50	+0.89	(−3.02, +4.79)	0.212	0.00
	0.75	+0.85	(−2.44, +4.14)	0.188	0.02

All eight ϱ-specific microperimetry analyses remained nonsignificant. A correlation of ϱ = 0 was used in the primary HORIZON analysis because the within-participant correlation was not reported.

MD, mean difference; dB, decibels; ETDRS, Early Treatment Diabetic Retinopathy Study; DL, DerSimonian–Laird; HKSJ, Hartung–Knapp–Sidik–Jonkman.

Programs: XIRIUS Part 1 (Cehajic-Kapetanovic 2020 / von Krusenstiern 2023), XIRIUS Part 2 (Lam 2024), Botaretigene trial (Michaelides 2024), HORIZON (Yang 2025). Random-effects point estimates and between-cohort variance were estimated using the DerSimonian–Laird method. Primary inference used Hartung–Knapp–Sidik–Jonkman small-sample intervals; conventional normal-approximation (z-based) DerSimonian–Laird intervals were reported as sensitivity analyses.

[a] Primary analyses used one estimate per independent cohort. XIRIUS Part 1 used observed participant-level paired changes; XIRIUS Part 2 combined treated arms and counted the shared control once.

[b] Arm-level estimates are descriptive because contrasts sharing a control are not independent.

[c] Leave-one-cohort-out analyses did not identify a dominant cohort.

[d] HORIZON sampling variances were recalculated for assumed correlations of ϱ = 0–0.75. XIRIUS Part 1 used observed paired differences and required no assumed correlation.

[e] Normal-approximation DL intervals are sensitivity analyses; HKSJ is the primary inference.

[f] Reliability sensitivity excluded XIRIUS Part 1 participant C5.3, whose acquisition was described as unreliable; the pooled estimate was essentially unchanged.

Table S8. Heterogeneity-estimator and robustness sensitivity analyses for the pooled functional outcomes.

Outcome	Method	τ^2	Poole d MD	95% CI	P
Microperimetry, 6 mo	Primary (DerSimonian–Laird + HKSJ)	0.18	+1.11	−0.88, +3.09	0.138
	REML + HKSJ	0.00	+1.06	−0.65, +2.78	0.117
	Paule–Mandel + HKSJ	0.27	+1.12	−0.93, +3.18	0.143
	Profile-likelihood b	—	+1.06	+0.24, +2.10	—
	95% prediction interval c	—	—	−6.61, +8.82	—
Microperimetry, 12 mo	Primary (DerSimonian–Laird + HKSJ)	0.00	+0.97	−3.99, +5.94	0.243
	REML + HKSJ	0.00	+0.97	−3.99, +5.94	0.243
	Paule–Mandel + HKSJ	0.00	+0.97	−3.99, +5.94	0.243
	Profile-likelihood b	—	+0.97	−0.02, +2.37	—
	95% prediction interval c	—	—	Not estimable ($k - 2 = 0$)	—
BCVA, 6 mo	Primary (DerSimonian–Laird + HKSJ)	3.62	+0.94	−4.97, +6.85	0.565
	REML + HKSJ	3.62	+0.94	−4.97, +6.85	0.565
	Paule–Mandel + HKSJ	3.62	+0.94	−4.97, +6.85	0.565
	Profile-likelihood b	—	+0.93	−2.18, +4.08	—
	95% prediction interval c	—	—	−28.87, +30.75	—

MD, mean difference; τ^2, between-study variance; HKSJ, Hartung–Knapp–Sidik–Jonkman. Point estimates were similar across variance estimators; all primary HKSJ intervals crossed the null.

[b] Profile-likelihood intervals omit the small-sample t correction. The Month-6 microperimetry interval excluded the null, indicating model dependence rather than a separate confirmatory result.

[c] Prediction intervals were wide and crossed the null; the Month-12 interval was not estimable.

Dependence and leave-one-cohort-out analyses are reported in Table S7.

Table S9. Trial-level ocular adverse-event counts underlying the aggregate safety proportions (central-injection cohorts).

Adverse event	XIRIUS Part 1 (n = 18)	XIRIUS Part 2 (n = 23)	Botaretigene (n = 32)	HORIZON central (n = 21)	Aggregate	Exact % (95% CI)
Any ocular serious adverse event	5/18	6/23	2/32	3/21	16/94	17.0 (10.1–26.2)
Procedure-related retinal complication	2/18	4/23	1/32	1/21	8/94	8.5 (3.7–16.1)
Retinal detachment	1/18	1/23	1/32	1/21	4/94	4.3 (1.2–10.5)
Intraocular inflammation (participant-level composite)	12/18	22/23	25/32	Not derivable	59/73	80.8 (69.9–89.1)
Anterior chamber cell (reported component term) †	3/18	NR	16/32	5/21	24/71	33.8 (23.0–46.0)
Elevated IOP / ocular hypertension (reported component term) †	6/18	NR	16/32	NR	22/50	44.0 (30.0–58.7)

Values are participants with at least one event / participants treated; NR, not reported. Exact Clopper–Pearson aggregate estimates are primary; model-based sensitivity analyses are reported in Table S6.

† Component-outcome denominators include only cohorts reporting explicit participant-level counts; nonreporting cohorts were excluded. HORIZON did not report a deduplicated inflammation composite, and its component terms were not summed.

Excluding one XIRIUS Part 1 ocular SAE attributed to natural disease progression yielded 15/94 (16.0%; 95% CI, 9.2–25.0). Retinal detachment is included within procedure-related retinal complications.

Table S10. Administered-dose documentation and per-eye calculations

Programme (cohort or participant)	Reported dose expression	Vector concentration	Injected volume	Dose per eye	Reported or calculated	Delivery route	Analytic context
XIRIUS Part 1, C1.1 (cohort 1)	Concentration and volume	5×10^{10} gp/mL	0.10 mL	5×10^{9} gp	Calculated ($5\times10^{10} \times 0.10$)	Central	Efficacy + safety
XIRIUS Part 1, C1.2 (cohort 1)	Concentration and volume	5×10^{10} gp/mL	0.10 mL	5×10^{9} gp	Calculated ($5\times10^{10} \times 0.10$)	Central	Efficacy + safety
XIRIUS Part 1, C1.3 (cohort 1)	Concentration and volume	5×10^{10} gp/mL	0.04 mL	2×10^{9} gp	Calculated ($5\times10^{10} \times 0.04$)	Central	Efficacy + safety
XIRIUS Part 1, C2.1 (cohort 2)	Concentration and volume	1×10^{11} gp/mL	0.04 mL	4×10^{9} gp	Calculated ($1\times10^{11} \times 0.04$)	Central	Efficacy + safety
XIRIUS Part 1, C2.2 (cohort 2)	Concentration and volume	1×10^{11} gp/mL	0.10 mL	1×10^{10} gp	Calculated ($1\times10^{11} \times 0.10$)	Central	BCVA + safety; no microperimetry
XIRIUS Part 1, C2.3 (cohort 2)	Concentration and volume	1×10^{11} gp/mL	0.10 mL	1×10^{10} gp	Calculated ($1\times10^{11} \times 0.10$)	Central	BCVA + safety; no microperimetry
XIRIUS Part 1, C3.1 (cohort 3)	Concentration and volume	5×10^{11} gp/mL	0.10 mL	5×10^{10} gp	Calculated ($5\times10^{11} \times 0.10$)	Central	Efficacy + safety
XIRIUS Part 1, C3.2 (cohort 3)	Concentration and volume	5×10^{11} gp/mL	0.03 mL	1.5×10^{10} gp	Calculated ($5\times10^{11} \times 0.03$)	Central	Efficacy + safety
XIRIUS Part 1, C3.3 (cohort 3)	Concentration and volume	5×10^{11} gp/mL	0.10 mL	5×10^{10} gp	Calculated ($5\times10^{11} \times 0.10$)	Central	Efficacy + safety
XIRIUS Part 1, C4.1 (cohort 4)	Concentration and volume	1×10^{12} gp/mL	0.06 mL	6×10^{10} gp	Calculated ($1\times10^{12} \times 0.06$)	Central	Efficacy + safety
XIRIUS Part 1, C4.2 (cohort 4)	Concentration and volume	1×10^{12} gp/mL	0.10 mL	1×10^{11} gp	Calculated ($1\times10^{12} \times 0.10$)	Central	Efficacy + safety
XIRIUS Part 1, C4.3 (cohort 4)	Concentration and volume	1×10^{12} gp/mL	0.10 mL	1×10^{11} gp	Calculated ($1\times10^{12} \times 0.10$)	Central	Safety and BCVA; fellow-eye Month-6 microperimetry invalid
XIRIUS Part 1, C5.1 (cohort 5)	Concentration and volume	2.5×10^{12} gp/mL	0.15 mL	3.75×10^{11} gp	Calculated ($2.5\times10^{12} \times 0.15$)	Central	Efficacy + safety
XIRIUS Part 1, C5.2 (cohort 5)	Concentration and volume	2.5×10^{12} gp/mL	0.10 mL	2.5×10^{11} gp	Calculated ($2.5\times10^{12} \times 0.10$)	Central	Efficacy + safety
XIRIUS Part 1, C5.3 (cohort 5)	Concentration and volume	2.5×10^{12} gp/mL	0.05 mL	1.25×10^{11} gp	Calculated ($2.5\times10^{12} \times 0.05$)	Central	Contributed; microperimetry described as unreliable (reliability sensitivity, Table S7)
XIRIUS Part 1, C6.1 (cohort 6)	Concentration and volume	5×10^{12} gp/mL	0.08 mL	4×10^{11} gp	Calculated ($5\times10^{12} \times 0.08$)	Central	Efficacy + safety
XIRIUS Part 1, C6.2 (cohort 6)	Concentration and volume	5×10^{12} gp/mL	0.05 mL	2.5×10^{11} gp	Calculated ($5\times10^{12} \times 0.05$)	Central	Efficacy + safety
XIRIUS Part 1, C6.3 (cohort 6)	Concentration and volume	5×10^{12} gp/mL	0.03 mL	1.5×10^{11} gp	Calculated ($5\times10^{12} \times 0.03$)	Central	Efficacy + safety
XIRIUS Part 2, low dose (Lam 2024)	Dose per eye	Not applicable	Reported per eye	5×10^{10} vg	Reported directly	Central	Efficacy + safety
XIRIUS Part 2, high dose (Lam 2024)	Dose per eye	Not applicable	Reported per eye	2.5×10^{11} vg	Reported directly	Central	Efficacy + safety
Botaretigene, low dose (Michaelides 2024)	Concentration; overall volume summary only	1.0×10^{11} vg/mL	0.50 mL (overall median; range 0.10–0.80; mean 0.42)	$\approx5\times10^{10}$ vg	Approximate; calculated using the overall median injected volume; participant-level dose not derivable	Central	Efficacy + safety
Botaretigene, intermediate dose	Concentration; overall volume summary only	2.0×10^{11} vg/mL	0.50 mL (overall median)	$\approx1\times10^{11}$ vg	Approximate; calculated using the overall median injected volume; participant-level dose not derivable	Central	Efficacy + safety
Botaretigene, high dose	Concentration; overall volume summary only	4.0×10^{11} vg/mL	0.50 mL (overall median)	$\approx2\times10^{11}$ vg	Approximate; calculated using the overall median injected volume; participant-level dose not derivable	Central	Contributed to safety; did not contribute to the pooled BCVA estimate
HORIZON, Group 1 (Yang 2025)	Dose per eye	Not applicable	Up to 0.3 mL	2.48×10^{10} vg	Reported directly	Mixed: 1 central, 3 peripheral	1 centrally injected participant contributed to the primary central-injection safety analysis; the 3 peripherally injected participants contributed only to the all-groups safety sensitivity analysis. Neither contributed to the efficacy pool.
HORIZON, Group 2	Dose per eye	Not applicable	Up to 0.3 mL	7.45×10^{10} vg	Reported directly	Mixed: 2 central, 2 peripheral	2 centrally injected participants contributed to the primary central-injection safety analysis; the 2 peripherally injected participants contributed only to the all-groups safety sensitivity analysis. Neither contributed to the efficacy pool.
HORIZON, Group 3	Dose per eye	Not applicable	Up to 0.3 mL	2.24×10^{11} vg	Reported directly	Peripheral (n = 3)	All-groups safety only
HORIZON, Group 4	Dose per eye	Not applicable	Up to 0.3 mL	2.24×10^{11} vg	Reported directly	Central (n = 7)	Efficacy + safety
HORIZON, Group 5	Dose per eye	Not applicable	Up to 0.3 mL	6.8×10^{11} vg	Reported directly	Central (n = 7)	Efficacy + safety

HORIZON, Group 6	Dose per eye	Not applicable	Up to 0.3 mL	1.99×10^{12} vg	Reported directly	Central (n = 4)	Safety only; above MTD, excluded from efficacy
LUMEOS, RPGR2e11 group (registry record)	Concentration and volume range	2.0×10^{11} vg/mL	0.30 – 0.80 mL per eye	6×10^{10} – 1.6×10^{11} vg	Calculated as a range (2.0×10^{11} × 0.30–0.80); participant-level dose not derivable	Central, bilateral	Narrative only
LUMEOS, RPGR4e11 group (registry record)	Concentration and volume range	4.0×10^{11} vg/mL	0.30 – 0.80 mL per eye	1.2×10^{11} – 3.2×10^{11} vg	Calculated as a range (4.0×10^{11} × 0.30–0.80); participant-level dose not derivable	Central, bilateral	Narrative only

Notes. XIRIUS Part 1 doses were calculated as concentration × injected volume; XIRIUS Part 2 and HORIZON reported dose per eye. Botaretigene values are approximate because only study-level volume summaries were available, and LUMEOS ranges are shown for narrative documentation only. Units were retained as reported (gp or vg) and were not interconverted. Dose was interpreted only within each program; no cross-platform equivalence or dose–response inference was made.

Source locations. XIRIUS Part 1 participant-level concentration, injected volume, and administered dose: Cehajic-Kapetanovic 2020, Table 1. XIRIUS Part 2 dose per eye: Lam 2024, Methods (study design and treatment). Botaretigene concentrations and injected-volume summary: Michaelides 2024, Methods and Safety results. HORIZON group doses, delivery routes, and maximum injected volume: Yang 2025, Treatment section and Figure 1. LUMEOS concentrations and volume ranges: NCT04671433/NL-OMON52296 registry intervention records.

gp, genome particles; vg, vector genomes; MTD, maximum tolerated dose; BCVA, best-corrected visual acuity.

Table S11. Comparator-stratified cohort estimates and external-control exclusion.

Comparator design is shown for transparency. Formal comparator subgroup analyses were not performed because each outcome-by-comparator stratum contained one cohort, except the two-cohort fellow-eye stratum at Month 6.

Outcome / timepoint	Cohort	Comparator type	Cohort-level estimate	In overall pool	Design handling
Microperimetry, 6 mo	XIRIUS Part 1	Untreated fellow eye	+0.43 dB	Yes	Observed paired change; no assumed correlation
	HORIZON	Untreated fellow eye	+0.97 dB	Yes	Paired-eye sensitivity (Table S7)
	XIRIUS Part 2	Concurrently randomized untreated	+2.33 dB	Yes	Independent concurrent control
Microperimetry, 12 mo	HORIZON	Untreated fellow eye	+0.78 dB	Yes	Paired-eye sensitivity (Table S7); exploratory
	XIRIUS Part 2	Concurrently randomized untreated	+1.75 dB	Yes	Independent control; exploratory
BCVA, 6 mo	XIRIUS Part 1	Untreated fellow eye	−0.89 letters	Yes	Observed paired change; no assumed correlation
	XIRIUS Part 2	Concurrently randomized untreated	+0.11 letters	Yes	Independent concurrent control
	Botaretigene	Deferred-treatment control (mixed design) [a]	+3.64 letters	Yes	Mixed design; control concurrent through Week 26
LLVA responder, 6 mo	XIRIUS Part 1	Untreated fellow eye	Responder contrast (Figure S1A)	Yes	Paired; exploratory
	XIRIUS Part 2	Concurrently randomized untreated	Responder contrast (Figure S1A)	Yes	Independent; exploratory
LLVA responder, 12 mo	XIRIUS Part 1	Untreated fellow eye	Responder contrast (Figure S1B)	Yes	Paired; exploratory
	XIRIUS Part 2	Concurrently randomized untreated	Responder contrast (Figure S1B)	Yes	Independent; exploratory
Context only	XOLARIS	External natural history	—	No	Narrative context only; excluded from all quantitative estimates [b]

Estimates are treated-minus-control mean differences from Figures 2–3; dB for microperimetry and ETDRS letters for BCVA.

[a] Botaretigene pooled nonrandomized escalation and randomized expansion participants against deferred controls through Week 26 and is therefore classified as a mixed-design comparison.

[b] External XOLARIS comparisons reported by von Krusenstiern were excluded; only untreated fellow-eye contrasts were synthesized.

Table S12. Microperimetry endpoint-compatibility assessment for quantitative synthesis

Only mesopic MAIA 68-locus 10-2 acquisitions were pooled. Static or scotopic perimetry and incompatible grids were retained for narrative synthesis only.

Cohort	Device	Luminance	Grid / stimulus set	Endpoint (units)	In microperimetry pool
XIRIUS Part 1	MAIA (CenterVue)	Mesopic	10-2, 68 stimuli, central 10°	Mean sensitivity (dB)	Yes
XIRIUS Part 2	MAIA (CenterVue)	Mesopic	10-2, 68 stimuli, central 10°	Mean sensitivity (dB)	Yes
HORIZON	MAIA (CenterVue)	Mesopic	10-2, 68 loci; whole-grid mean	Mean sensitivity (dB)	Yes
Botaretigene	Static perimetry (primary); scotopic microperimetry	Photopic (static) / scotopic	Static 10° grid; scotopic MP	Static / scotopic MRS	No [a]
LUMEOS (phase 3 registry-results report)	Static perimetry	Photopic (static)	Static perimetry grid; no MAIA 10-2 68-locus acquisition reported	Mean retinal sensitivity (dB); binocular vision-guided mobility as primary endpoint	No [b]

MAIA, Macular Integrity Assessment; MRS, mean retinal sensitivity; dB, decibels.

[a] Botaretigene used static and scotopic testing and contributed to BCVA, but not to the mesopic MAIA pool.

[b] LUMEOS used static perimetry and was retained for narrative synthesis only.

Reliability and data handling by cohort.

Reliability procedures differed between trials and were considered in the risk-of-bias assessment.

Cohort	Reliability / data handling reported
XIRIUS Part 1	Baseline microperimetry was performed in triplicate over two days, with the third reading used as baseline. One participant (C5.3) had an unreliable acquisition; its handling in the aggregate summary was not fully reported.
XIRIUS Part 2	Assessments masked from Month 3; missing loci addressed by multiple imputation and repeated-measures modeling.
HORIZON	MAIA fundus tracking; responder status required confirmation at two consecutive visits.
Botaretigene	Not applicable to the mesopic MAIA pool.
LUMEOS	Static perimetry; narrative synthesis only.

Table S13. Protocol amendments and post-registration implementation decisions

These amendments did not alter the review population, intervention, comparator, eligibility criteria, quantitative-analysis study set, or prespecified outcome domains. Their effects on synthesis and interpretation are summarized below.

Item	Registered plan (PROSPERO v1.0)	As conducted	Classification	Timing	Rationale	Effect on synthesis and interpretation
**Search strategy and update**	Multi-source search described with a single free-text strategy; end date 31 January 2026.	The original search was retained and documented by platform. Searches were rerun with platform-specific syntax before resubmission.	Reporting update	Manuscript preparation; before resubmission	To provide reproducible strategies and a current search.	LUMEOS was added to the narrative synthesis; the quantitative-analysis study set was unchanged.
**Microperimetry endpoint compatibility**	Retinal-sensitivity synthesis using standardized instruments (e.g., MAIA, Nidek, or comparable).	Quantitative pooling was restricted to mesopic MAIA 68-locus acquisitions; incompatible luminance conditions, devices, and grids were retained for narrative synthesis.	Outcome-compatibility decision	Data extraction; before quantitative synthesis	Pooling noncomparable retinal-sensitivity frameworks would weaken interpretability.	Restricted the data entering the microperimetry pool without changing included studies or outcome domains.
**Shared controls, non-independence, and paired-eye dependence**	Random-effects meta-analysis; no explicit plan for shared controls or paired-eye dependence.	Primary analyses used one estimate per independent cohort. Multi-arm treated groups were combined and shared controls counted once. XIRIUS Part 1 used observed paired changes; HORIZON used rho = 0-0.75 sensitivity analyses (Table S7).	Analytic correction	Quantitative synthesis; revised before resubmission	Shared-control contrasts are dependent, and observed pairing should be used when available.	Intervals widened and Month-12 microperimetry was designated exploratory because only two cohorts contributed. Recalculation of XIRIUS Part 1 changed the Month-6 microperimetry and BCVA point estimates; primary conclusions and GRADE ratings were unchanged.
**Primary inference for continuous outcomes**	Random-effects models using DerSimonian-Laird or REML.	DerSimonian-Laird estimates with HKSJ intervals were used for primary inference; conventional and alternative estimators were retained as sensitivity analyses.	Analytic refinement	Quantitative synthesis	Normal-approximation intervals can be anticonservative when few studies contribute.	Affected confidence intervals and inference, but not included studies or extracted effect estimates.
**Aggregate safety synthesis**	Single-arm proportions synthesized with a random-effects model.	Exact Clopper-Pearson proportions were used as the primary description, with random-effects logit models as sensitivity analyses. Central-injection cohorts formed the primary analysis; all groups were examined in sensitivity analyses.	Analytic refinement	Revision, before resubmission	Exact intervals remain valid for sparse aggregate counts; model-based analyses retain between-cohort context.	Changed interval estimation only; event counts and contributing studies were unchanged.
**Definition of ocular adverse-event outcomes**	Safety defined as the frequency and severity of ocular serious adverse events and any ocular adverse events during follow-up.	Safety was reported as any ocular SAE, procedure-related retinal complication, retinal detachment, and deduplicated intraocular inflammation; anterior chamber cell and IOP/OHT were descriptive components.	Outcome-definition refinement	Data extraction and revision	A global count mixes clinically distinct events and overlapping preferred terms.	Changed safety reporting and GRADE categorization; nonderivable categories were not treated as zero.
**Risk-of-bias method for non-comparative safety outcomes**	Risk of bias assessed with a risk-of-bias tool; no framework specified for uncontrolled harm proportions.	A review-specific, nonvalidated five-domain framework was specified before reassessment of non-comparative safety outcomes (Table S2).	Methodological refinement	Revision, before safety reassessment	Comparative risk-of-bias tools do not evaluate uncontrolled absolute event proportions.	Changed the risk-of-bias method only; sparse data remained under GRADE imprecision.
**Administered-dose and delivery context**	Exploratory subgroup analyses by dose, vector serotype, and follow-up duration.	Dose was retained as descriptive within-program context. The above-MTD cohort was shown separately and excluded from pooled efficacy. Formal cross-program dose comparisons were not performed.	Interpretive refinement	Data extraction and revision	Dose is confounded with vector platform, and equal vg/eye values are not equipotent across programs.	Changed presentation and interpretation of dose without changing included studies or outcome domains.
**Non-comparable responder definitions**	Responder proportions to be synthesized where reported.	Microperimetry responder definitions were judged noncomparable and summarized descriptively rather than pooled.	Outcome-compatibility decision	Data extraction; before quantitative synthesis	Definitions differed in grid, threshold, luminance condition, and confirmation requirements.	The outcome was not pooled; included studies were unchanged.
**LLVA responder synthesis**	Responder outcomes to be synthesized where feasible.	The low-luminance responder outcome was synthesized with a Mantel-Haenszel risk ratio without continuity correction and interpreted as exploratory.	Analytic clarification	Quantitative synthesis	Sparse events and two contributing studies limit stable inference.	Changed analysis and interpretation only; included studies were unchanged.
**GRADE implementation**	Certainty to be assessed using GRADE, with GRADEpro GDT as supporting software.	GRADE was applied manually using ROBINS-I-integrated guidance; domain rationales are reported in Table S3.	Implementation refinement	Revision	Standard GRADEpro templates did not accommodate all contributing designs.	Documentation and software only; no certainty rating changed.
**Statistical software**	RevMan for parallel-arm meta-analysis and R packages for single-arm and advanced analyses.	Python 3.12.3 with NumPy, SciPy, and Matplotlib was used throughout.	Software substitution	Before quantitative synthesis	A single reproducible pipeline supported all primary and sensitivity analyses.	Software substitution only; data and estimands were unchanged. Code is available in the Zenodo deposit.

MTD, maximum tolerated dose; LLVA, low-luminance visual acuity; BCVA, best-corrected visual acuity; IOP/OHT, intraocular pressure/ocular hypertension; SAE, serious adverse event. Timing refers to the review stage. The contemporaneous PROSPERO version 1.0 record (CRD420251163589; 9 October 2025) was retained as the original registered plan.

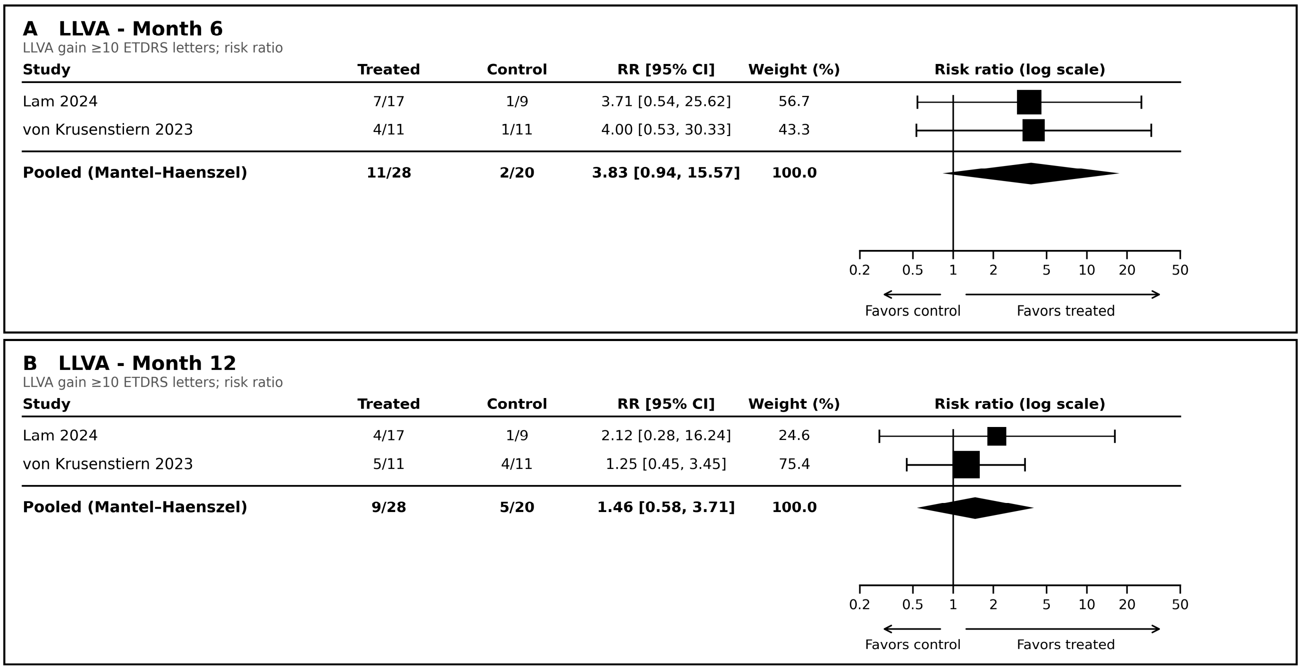

Figure S1. Low-luminance visual acuity responder analysis. Proportion of eyes gaining ≥10 ETDRS letters of low-luminance acuity, treated versus untreated, pooled as risk ratios. (A) At 6 months, the estimate favored treatment but was imprecise and not statistically significant under the primary risk-ratio analysis (RR 3.83, 95% CI 0.94-15.57). (B) By 12 months, responder proportions were more similar between treated and untreated eyes (RR 1.46, 95% CI 0.58-3.71).
